# T-Switch: A specificity-based engineering platform for developing safe and effective T cell therapeutics

**DOI:** 10.1016/j.immuni.2024.11.009

**Published:** 2024-12-03

**Authors:** Nouran S. Abdelfattah, Tomasz Kula, Stephen J. Elledge

**Affiliations:** 1 Department of Genetics, Harvard Medical School, Division of Genetics, Brigham and Women’s Hospital, Howard Hughes Medical Institute, 77 Avenue Louis Pasteur, Boston, MA 02115, USA; 2 Lead contact

## Abstract

Many promising targets for adoptive T cell therapy (ACT) are self-antigens, but self-reactive T cells are generally eliminated during thymic selection or diverted to regulatory phenotypes. To bypass T cell tolerance and obtain potent and safe T cell therapeutics, we developed T-Switch, an *in vitro* T cell receptor (TCR) engineering platform for the creation, modification, and comprehensive profiling of TCRs that can target self-antigens. T-Switch first expands T cells that recognize a “foreign” peptide closely related to a self-antigen. The fine specificity of the TCR is then modified by directed evolution of the peptide binding region to switch its specificity to the self-antigen of interest. We applied T-Switch to engineer synthetic TCRs reactive to a tumor-associated self-antigen, validated the safety and efficacy of this approach, and detected no off-target recognition as measured against the human proteome. Thus, T-Switch represents a resource for the creation of collections of highly sensitive synthetic TCRs for T cell-based immunotherapies.

## In brief

Abdelfattah et al. developed an engineering platform (T-Switch) for the creation, modification, and high-throughput profiling of T cell receptors (TCRs) to desired targets, particularly self-antigens. T-Switch identified potent and specific candidate TCRs against a clinically relevant cancer self-target highly expressed in neuroblastoma and has potential for broader application in immunotherapy.

## INTRODUCTION

T cells play critical roles in cellular immunity, including the elimination of cancer cells with tumor-associated antigens. T cell receptors (TCRs) expressed on T cells mediate this action by recognition of peptides bound to major histocompatibility complex (MHC) molecules.^1–3^ Advances in immunotherapy utilizing TCRs have led to the development of adoptive T cell therapy (ACT). ACT involves the isolation and genetic modification of patient-derived T cells to enhance their anti-tumor activity by introducing cancer-specific TCRs or a chimeric antigen receptor (CAR), followed by their *ex vivo* expansion and infusion back into the cancer patient.^4–8^ An advantage of using TCRs over CARs is their ability to recognize internal antigens at very low antigen densities on tumors, expanding the breadth of antigens that can be targeted.

Internal antigens are categorized into neoantigens and tumor-associated self-antigens^4^; the former are unique to each patient, necessitating personalized therapies, while the latter can be common across cancer patients, allowing for more standardized treatments. Many self-antigens, like MAGE-A1, MAGE-A3, and NY-ESO-1, encode antigens overexpressed in tumor cells across cancers^5^ and are primarily expressed in germline cells that lack human leukocyte antigen (HLA) molecules and therefore do not present antigens to T cells.^6^ Other antigens include cell lineage differentiation antigens, such as gp100, MART-1,^7^ tyrosinase,^8^ and prostate-specific antigen (PSA),^9,10^ that are normally not expressed in adult tissues.^11^ Tissue-restricted antigens expressed in cancer cells and certain non-essential tissues, such as melanocytes, breast epithelia, testes, fallopian tubes, and ovaries, may serve as promising therapeutic targets.

Several clinical efforts of TCR-engineered T cells targeting self-antigens have demonstrated objective responses in patients with refractory or relapsed solid tumors.^12–15^ However, targeting tumor self-antigens is complicated by central tolerance, which involves the negative selection of developing T cells that recognize and react with high affinity to self-antigens in the thymus. For several tissue-restricted genes, their expression by medullary thymic epithelial cells leads to T cell tolerance.^16^ Consequently, naturally occurring TCRs with high affinity to self-antigens are extremely rare.

To improve the properties of low-affinity TCRs to self-antigens, directed evolution and protein engineering methods have been employed to enhance TCR affinity to these antigens.^17,18^ One notable example is an HLA-A1-restricted TCR that targeted MAGE-A3, which was affinity-enhanced within the physiological range from ~500 to 2.3 μM. However, this TCR developed an off-target reactivity to the heart protein titin, resulting in cardiotoxicity and patient deaths.^11^ Moreover, it is now well appreciated that affinity enhancement alone does not correlate with improved TCR potency, and TCRs with high supraphysiological affinities (<1 μM) can lead to impaired function.^19–21^ Thus, the development of alternative engineering platforms that focus on engineering TCR specificity and sensitivity while simultaneously avoiding off-target promiscuity is needed. These approaches, coupled with more complete detection methods for cross-reactivity detection, would expand and accelerate the creation of T cell therapeutics. To address this, we developed T-Switch, an *in vitro* selection strategy aimed at facilitating small-step specificity changes, for the creation, modification, and high-throughput profiling of TCRs that represents an off-the-shelf platform for the potential discovery of highly potent TCRs against the large array of candidate internal antigens.

## RESULTS

### A directed evolution strategy to switch the specificity of two model NLV TCRs

**Figure 1.**
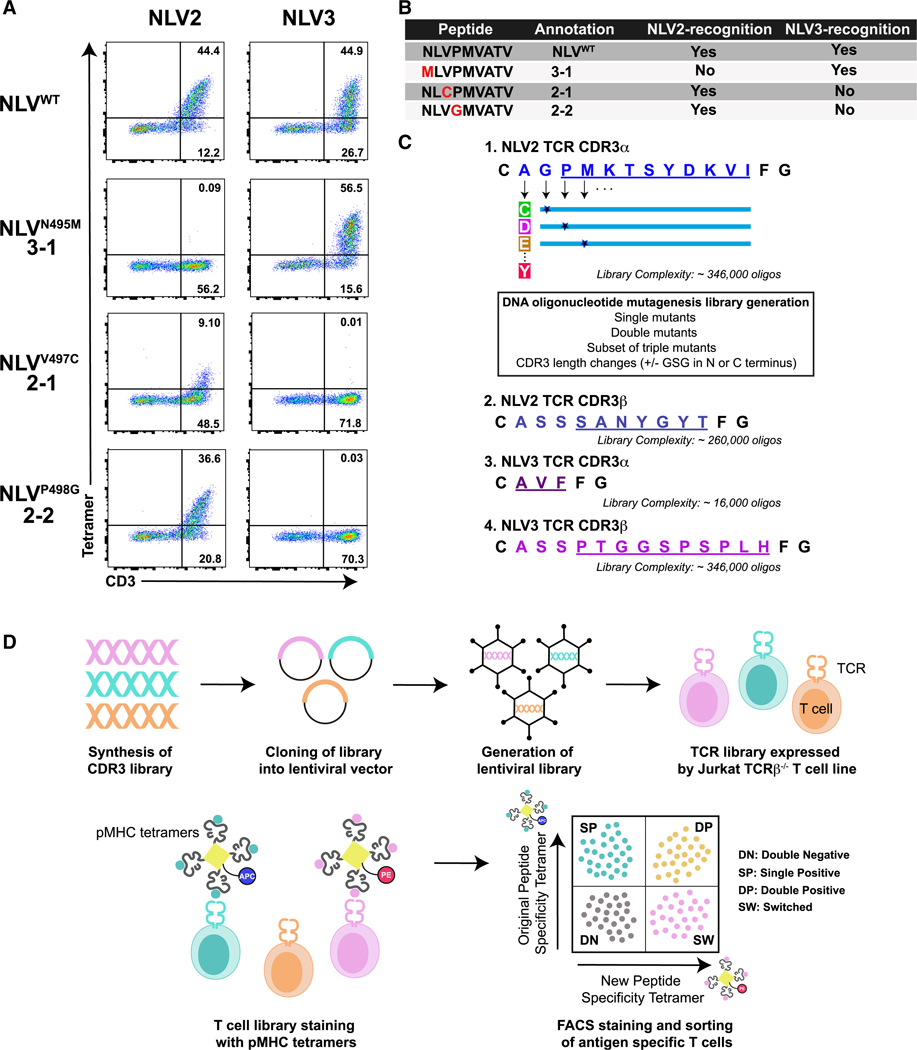
The design of comprehensive saturation mutagenesis libraries and selection strategy to switch the specificity of two model NLV TCRs (A) Jurkat TCRβ^−/−^ cells lentivirally infected with lentiviruses expressing the NLV2 TCR (left) or NLV3 TCR (right) were stained with tetramers containing variants of the pp65 peptide (NLVPMVATV). (B) Summary of tetramer staining properties illustrating differential peptide recognition of NLV2 and NLV3 TCRs. (C) Schematic detailing the design of CDR3 libraries. (D) Workflow of TCR screening strategy.

Our goal was to devise a method to generate TCRs to self-antigens, which are limited by central tolerance, while avoiding a loss of specificity. To achieve this, we reasoned that we could select a TCR to a neoantigen version of the self-peptide that differed in only a single residue, then mutagenize the TCR to reverse the specificity to the wild-type (WT) peptide. The swap in specificity should guarantee that the gained specificity was not simply through enhanced MHC interaction. Although changing TCR specificity to completely different peptides has been achieved,^22^ here we used directed evolution to systematically change the specificity of TCRs between closely related peptides. We initially employed two well-characterized viral TCRs: the NLV2 and NLV3 TCRs^23^ that recognize the HLA-A2-restricted cytomegalovirus (CMV) epitope (NLVPMVATV) or NLV for short (Figure 1A, top) chosen because they differentially recognize distinct one amino acid variants of the immunodominant NLV epitope.^24^ We selected some of these variants to validate via tetramer staining. Jurkat TCRβ^−/−^ cells lentivirally infected with NLV2 showed tetramer binding to an NLV^V497C^ (2–1) variant at position three of the epitope and an NLV^P498G^ (2–2) variant at position four of the epitope. Conversely, cells expressing NLV3 showed specificity to an NLV^N495M^ (3–1) variant at position one (Figures 1A and 1B). Having two unrelated TCRs that had overlapping but distinct specificities allowed us to ask the question as to whether we can switch the specificity from one epitope to another using directed evolution without altering their off-target profile.

To achieve the switch, we focused our library design on the complementary determining region 3 (CDR3) loop of the TCR. CDR3 is the major determinant of peptide specificity with the TCRα chain primarily contacting the N-terminal portion of the peptide and the TCRβ chain contacts the C-terminal portion.^25–28^ Given the mutation location, we expected the CDR3α chain libraries to drive the generation of novel specificities. To systematically and productively manipulate TCR specificity, we generated comprehensive saturation mutagenesis libraries that consist of all single mutants, all double mutants, a subset of triple mutants, and CDR3 length changes targeted to the CDR3α or β chains of the NLV2 and NLV3 TCRs (Figure 1C). We synthesized oligonucleotides encoding these libraries, which range from ~16,000–400,000 mutants depending on the size of the CDR3. CDR3 libraries were separately subcloned into an NLV2 or NLV3 TCR-expressing lentiviral backbone vector. The Jurkat TCRβ^−/−^ cell line was separately infected with each of the libraries at low multiplicity of infection (MOI) to generate a library of T cells with variant CDR3 loops. Antigen-specific TCR variants recognizing either the original epitope, the novel epitope, or both epitopes were isolated by tetramer staining and fluorescence-activated cell sorting (FACS) and characterized by next-generation sequencing (Figure 1D).

**Figure 2.**
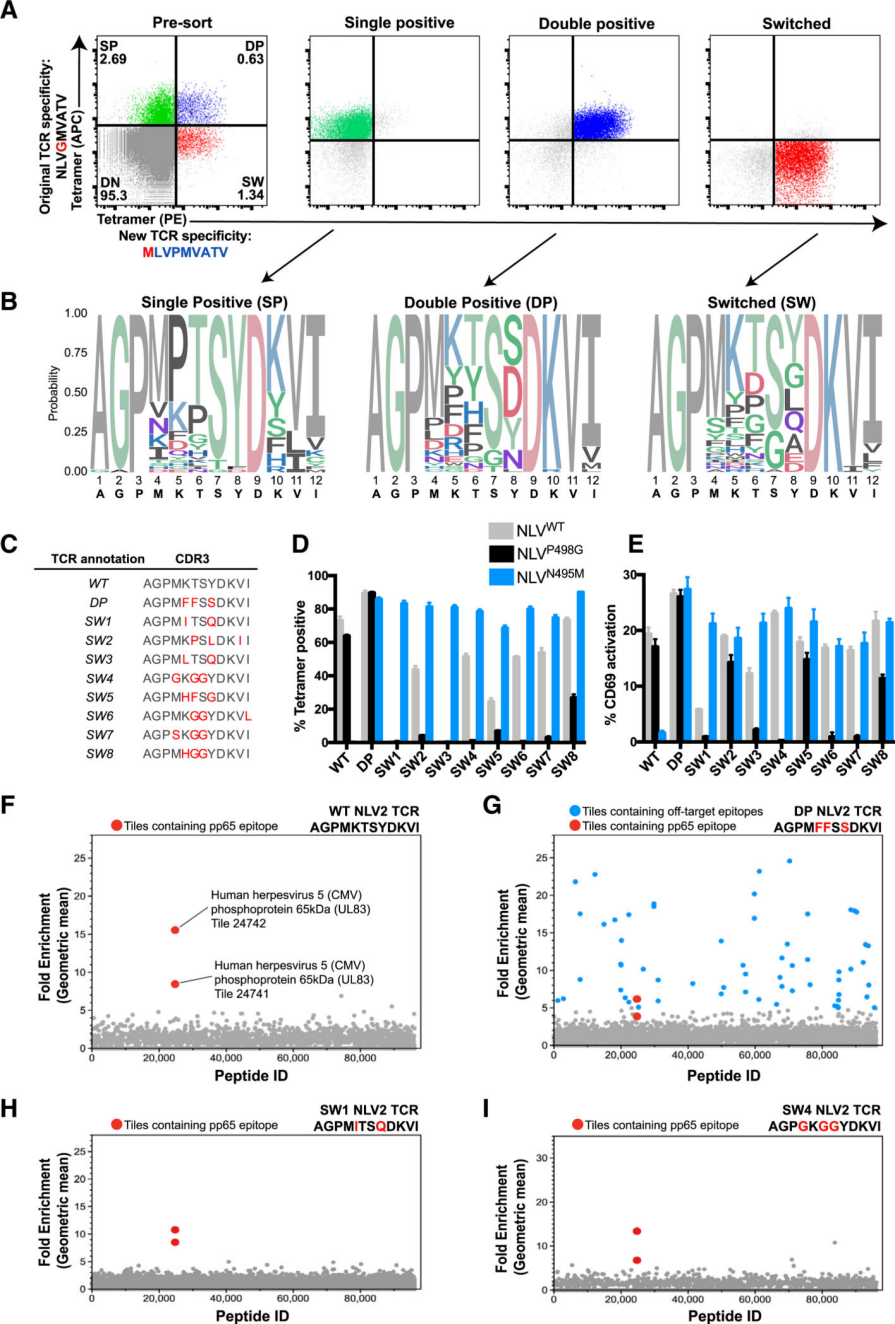
NLV2 CDR3α library screening and validation (A) Jurkat TCRβ^−/−^ cells infected with the library were stained and sorted using an NLV2-specific (NLVGMVATV) and an NLV3-specific (MLVPMVATV) peptide tetramer. All quadrants were sorted separately, and three (SP, DP, and SW) underwent one round of expansion prior to sequencing. (B) Amino acid sequence logos from the top 50 enriched CDR3α sequences show the frequency of specific residues at each CDR3α position across the different sorting populations. WT CDR3 residues are shown below the logo plot. (C) Sequences of TCRs selected for validation from each of the different sorting populations. Jurkat TCRβ^−/−^ cells were infected separately with lentiviral constructs expressing each of the TCRs. (D and E) Tetramer staining (D) and CD69 activation (E) for each of the validation TCRs shown as mean ± SDs of three technical replicates. (F–I) The cross-reactivity profiles of the NLV2 WT TCR (F), NLV2 DP TCR (G), and two switch TCRs (H and I) against the virome-wide library. Each dot represents one peptide tile. The y axis represents the geometric mean of the fold enrichment of the peptide across ten replicates (five screen replicates with two internal barcode replicates each). Peptides highlighted in red contain the known cognate antigen of the NLV2 TCRs, and those highlighted in blue contain off-target hits.

We first screened the NLV2 TCR library. The NLV2 α chain containing the CDR3α library (diversity: 346,000 oligos) was first paired with the WT NLV2 β chain in the transduced Jurkat cells. The library was then screened with peptide-MHC-loaded tetramers to identify variants that switched binding specificity from recognizing the NLV2-specific permissive mutant (2–2) to binding the NLV3-specific mutant (3–1). Greater than 95% of the library lost recognition of both peptides, leaving ~3% that retained binding to the NLV2-specific mutant, ~1% that bound both peptides, and ~1% that completely switched specificity to the NLV3-specific mutant (Figure 2A, left). Each population was sorted and expanded twice prior to next-generation sequencing (Figure 2A, right). The single positive (SP) population refers to the variant TCRs that retain binding to the original TCR specificity. The double positive (DP) population refers to the variants that bind to both the original specificity and the novel specificity, and the switched population (SW) signifies the TCR variants that lost specificity to the original NLV2 permissive binder and gained novel specificity to the NLV3-specific mutant.

Analysis of the allowable substitutions revealed certain patterns. CDR3 substitutions in the TCRs maintaining or improving binding to the original peptide (SP) showed permissivity for mutation of the lysines at positions 5 and 10 to more bulky residues, especially proline in position 5 and conservative substitutions at position 10 (Figures 2B and S1A; Table S1). Analysis of the DP and SW populations revealed mutations in novel CDR3 positions that do not overlap with the SP population, mainly a significant reliance on position 7 or 8 for recognition of the new binder (Figures 2B and S1A; Table S1). A key difference between the DP and SW populations is the presence of bulky hydrophobic residues at position 6 in the DP population that are rare in the SW population. Moreover, our library design allows us to dissect detrimental changes that demolish TCR binding to its original antigen. For example, single mutations at positions 4 and 5 tend to be permissive for binding the original antigen, while most mutations at the remaining positions tend to abolish recognition to the original binder (Figure S1B). We can also better understand the SP enrichments that enhance recognition to the original binder by normalizing the enrichments to that of the WT CDR3α enrichment (Figure S1C). For example, single amino acid substitutions of acidic residues at position 5 enhance recognition to the original binder (Figure S1C). All the SP enrichments overlap with the permissive mutations in Figure S1B, with the exception of the permissive mutants present in the DP population. This deep level of structure-function resolution could be useful in future studies to better dissect the determinants of TCR specificity. The second library of the NLV2 CDR3β chain paired with the WT NLV2 α chain did not show detectable DP or SW populations (Figure S1D), suggesting that the WT β chain CDR3 is not in contact with the N-terminal part of the peptide as expected from structural data.

The top eight enriched SW TCRs were selected for validation by binding and functional assays (Figure 2C). All SW NLV2 TCR-infected Jurkat cells acquired novel peptide (NLV^N495M^) recognition by tetramer binding (Figure 2D) and upregulated the early activation marker CD69 (Figure 2E) following co-culture with target cells pulsed with the NLV3-specific mutant. All TCRs retained activation to the NLV^WT^ immunodominant epitope that was not selected against during screening. Five of eight SW TCRs showed no cross-reaction to the NLV2-specific mutant (original binder), demonstrating a complete switch in specificity. Moreover, dose-response curves of three of the switched TCRs show that they all remain highly potent toward the NLV3-specific mutant and comparable to the WT NLV3 and NLV2 TCRs (Figure S1E). These experiments show that comprehensive mutagenesis libraries targeted to the CDR3 region of the TCR allow discrimination between closely related peptides and yield receptors with novel specificities and high potency.

### Comprehensive off-target profiling displays favorable specificity of NLV TCR switch variants

Each time a TCR is engineered, it poses a potential threat to its specificity profile. To gain a sense of whether we drastically altered the recognition profile of the TCRs while changing their specificity, we assessed their off-target reactivities via T-Scan.^24^ The advantage of the T-Scan method is its ability to interrogate large numbers of highly complex candidate antigens in a high-throughput screen. We selected the NLV2 WT TCR, a variant NLV2 TCR that can recognize both peptides (DP TCR) and two SW TCRs, expressed them in primary T cells, and screened them with the virome-wide library, which consists of 93,904 56 amino acid fragments that collectively tile across the proteomes of all viruses known to infect humans^24^ (Figure S2A). The library was transduced in cells that express only a single MHC, HLA-A2, in addition to the granzyme reporter. If the engineered TCR variants improved the non-specific recognition to the MHC itself, we would expect enhanced TCR recognition of multiple peptides on HLA-A2, similar to what was seen with the evolved MAGE-A3 TCR.^29^ As expected, the two most enriched peptides in the screen with the WT TCR were the only two tiles that contained the known NLV^WT^ epitope (Figure 2F; Table S2A). By contrast, the DP TCR recognized 16 additional off-targets, recognizing multiple peptides on HLA-A2 (Figure 2G; Tables S2B and S2C). To look closer at the recognition profile of the DP TCR, we used NetMHC to predict peptide binders from some of the scoring overlapping tiles that share similarity with the original peptide. We found that predicted off-target binders differ by up to 7 of 9 amino acid residues as compared with the original binder of the TCR. Given that these were the only predicted binding peptides in the 22 amino acid overlap regions of the scoring peptide tiles and were also the only regions that shared any similarity with the original peptide, this confirms the promiscuous nature of this TCR (Figure S2B). Conversely, the switched TCRs, one with two mutations in the CDR3α (SW1) and another with three mutations (SW4), both retained recognition of only the NLV^WT^ peptide tiles with no additional cross-reactivity (Figures 2H and 2I; Tables S2D and S2E). This phenomenon was further confirmed by performing additional virome-wide screens with a different NLV2-engineered DP variant (Figure S2C; Table S2F) and an SW3 variant (Figure S2D; Table S2G). Similarly, the second DP TCR^PHD^ acquired promiscuity, recognizing a different set of off-target tiles than those recognized with the first DP TCR^FFS^ screened (Figure S2C; Tables S2F and S3H). This suggests that the complete switch in specificity from a closely related epitope is key to avoid drastic changes to the off-target profile of the variant TCRs.

To further characterize the mutational fingerprint of the engineered TCRs, we performed epitope saturation mutagenesis T-Scan screens. To accomplish this, we generated a comprehensive single mutant library of both the 2–2 epitope variant and the 3–1 epitope variant, with each mutant epitope present in the context of both a 56-aa fragment and a 9-aa fragment (Figure S3A). As a control for the 56-aa version, we also mutagenized the two amino acids immediately upstream and downstream of the epitopes since they are not predicted to affect TCR recognition (Figure S3A). This mutant library was introduced into HLA-A2 T-Scan target cells and co-cultured with the primary T cells expressing the four different TCRs. To identify the critical binding residues, we assessed the relative enrichment of each mutant in comparison to the known or acquired binder of the selected TCR (Figures S3B–S3E). Specificity profiling of the NLV2 WT TCR revealed, as expected, that most mutations abrogated T cell killing, with the exception of a few relatively conservative substitutions at positions 2, 3, 4, and 6, which were tolerated (Figure S3B, left). Thus, patterns of residue changes that show enhanced T cell killing likely indicate some of the direct contact sites within the epitope. Since the NLV2 WT TCR does not bind the 3–1 peptide itself, the fingerprint of this TCR on the non-permissive 3–1 peptide prevented recognition at all substitutions, except the methionine to asparagine change at position 1, which reverted the sequence back to the primary NLV immunodominant epitope and revived recognition and killing (Figure S3B, right). Thus, these patterns are likely indicative of the precise contact sites within the epitope and gave us an opportunity to probe the extent of variation in the interface of the different engineered TCRs. The fingerprint of the DP TCR (DP1) was in stark contrast to that of the WT TCR, with extensive substitution permissivity at most positions for both fingerprints (Figure S3C). This was especially apparent at position 1, where the mutation lies, and it explains the ability of the DP TCR to recognize so many different epitopes in the virome-wide screen. For the SW TCR interfaces, we see that they acquired a very specific fingerprint on the novel 3–1 peptide and lost recognition at all positions to the 2–2 original binder (Figures S3D and S3E). Most mutations identified in the SW TCR interface appear to be chemically similar to the unmutated residue, thereby preserving the recognition profile of the SW TCRs.

**Figure 3.**
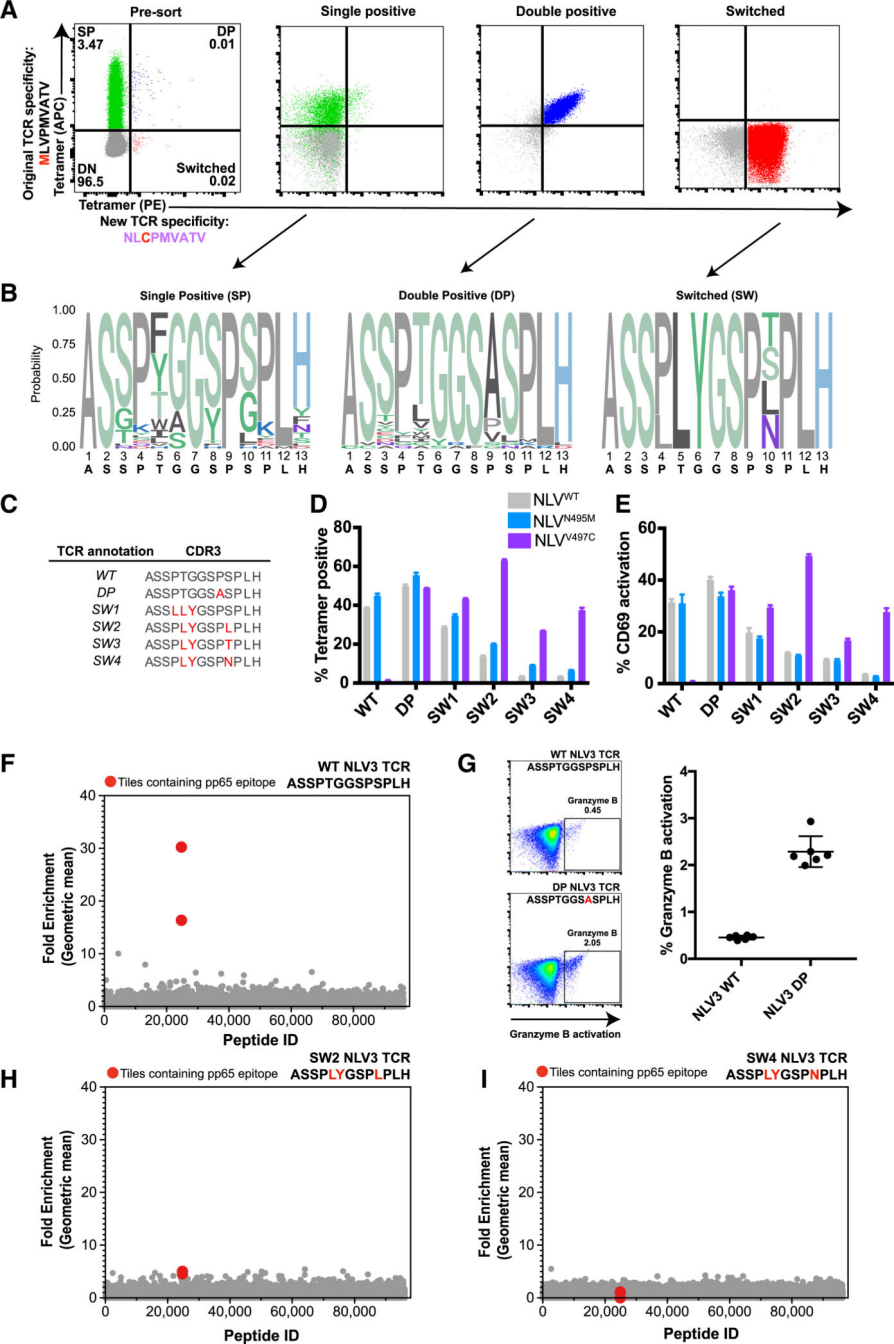
NLV3 CDR3β library screening and validation (A) The NLV3 CDR3β library was stained and sorted using an NLV3-specific peptide tetramer (MLVPMVATV) and an NLV2-specific peptide tetramer (NLCPMVATV) as described in Figures 2A and 2B. (B) Amino acid sequence logos showing the frequency of specific residues at each CDR3β position across as described in Figure 2B. (C) Sequences of the CDR3 sequences chosen for validation experiments. (D and E) Tetramer staining (D) and CD69 activation (E) for each of the validation TCRs expressed in Jurkat TCRβ^−/−^ cells. Data are shown as mean ± SDs of three technical replicates. (F–I) The cross-reactivity profiles of NLV3 WT (F) and two switch TCRs (H and I) against the virome-wide library as described in Figures 2F–2I. (G) Granzyme B reporter activation in T-Scan HLA-A2+ target cells after co-culture with primary T cells expressing either the WT NLV3 TCR (top) or the DP-engineered NLV3 TCR variant (bottom).

To examine the generality of this method, we similarly engineered a second NLV TCR: NLV3. The NLV3 TCR is unique because of its short CDR3α chain (only 3 amino acids) and its long CDR3β chain (13 amino acids long) (Figure 1C). This is interesting because the non-binding peptide we wish to evolve it to recognize (2–1) has its mutation in the N-terminal part of the peptide; therefore, we would expect to rely on the α chain to switch specificity. However, when we screened the NLV3 CDR3α chain library to switch its specificity from its original binder 3–1 to the closely related NLV2-specific peptide (2–1), no population enrichments were observed (Figure S4A). By contrast, after three rounds of sorting, the NLV3 CDR3β library identified four variant TCRs with re-directed specificity to the 2–1 peptide (Figures 3A, 3B, and S4B; Table S3). This suggests that the CDR3β chain of the NLV3 TCR is in contact with the N-terminal part of the peptide, in contrast to what is generally expected structurally from most TCR-epitope interactions. The four rare switch variants identified were triple mutants that contained an LY motif at positions 5 and 6, suggesting that we needed complex mutations for this switch in specificity. For the DP population, the major enrichment was a proline to alanine change at position 9 of the CDR3β chain either as a single mutant or coupled with a change at position 3 or 5 (Figures 3B and S4B). Validation of the SW TCR variants (Figure 3C) revealed binding (Figure 3D) and activation (Figure 3E) to the 2–1 peptide.

Next, we assessed the off-target landscape of these TCRs via virome-wide T-Scan. We selected four TCRs to screen: the WT NLV3 TCR, the single mutant proline to alanine DP variant, and two SW TCRs. Each of the TCRs was expressed separately in primary T cells and co-cultured with target T-Scan cells expressing the virome-wide library. As expected, the two most enriched peptides in the screen with the NLV3 WT TCR were the only two tiles that contained the known NLV^WT^ epitope (Figure 3F; Table S4A). Interestingly, co-culture of the DP TCR with the target cells revealed a high background of activation, so we co-cultured the WT or DP TCRs with HLA-A2 target cells without expression of the virome-wide library and tested for granzyme B reporter activation. We observed a high basal activation (~3%) of the reporter (Figure 3G), suggesting that the DP TCR has either acquired promiscuity to an antigen expressed in 293T cells or the engineering has enhanced the binding between the TCR and the HLA helices themselves. This extreme background activity precluded screening of this TCR. Importantly, the SW TCR screens revealed no additional off-targets or promiscuity (Figures 3H and 3I; Tables S4B and S4C), and the SW4 TCR also lost recognition to the WT immunodominant NLV (Figure 3I). Together, our results using two distinct TCRs show that simply gaining additional recognition in the DP population generates promiscuous TCRs and that the switch in specificity between closely related epitopes creates highly specific TCRs that are much more likely to generate safe and effective TCRs.

### Application of T-Switch for engineering TCRs with specificity and functionality to a tumor antigen

**Figure 4.**
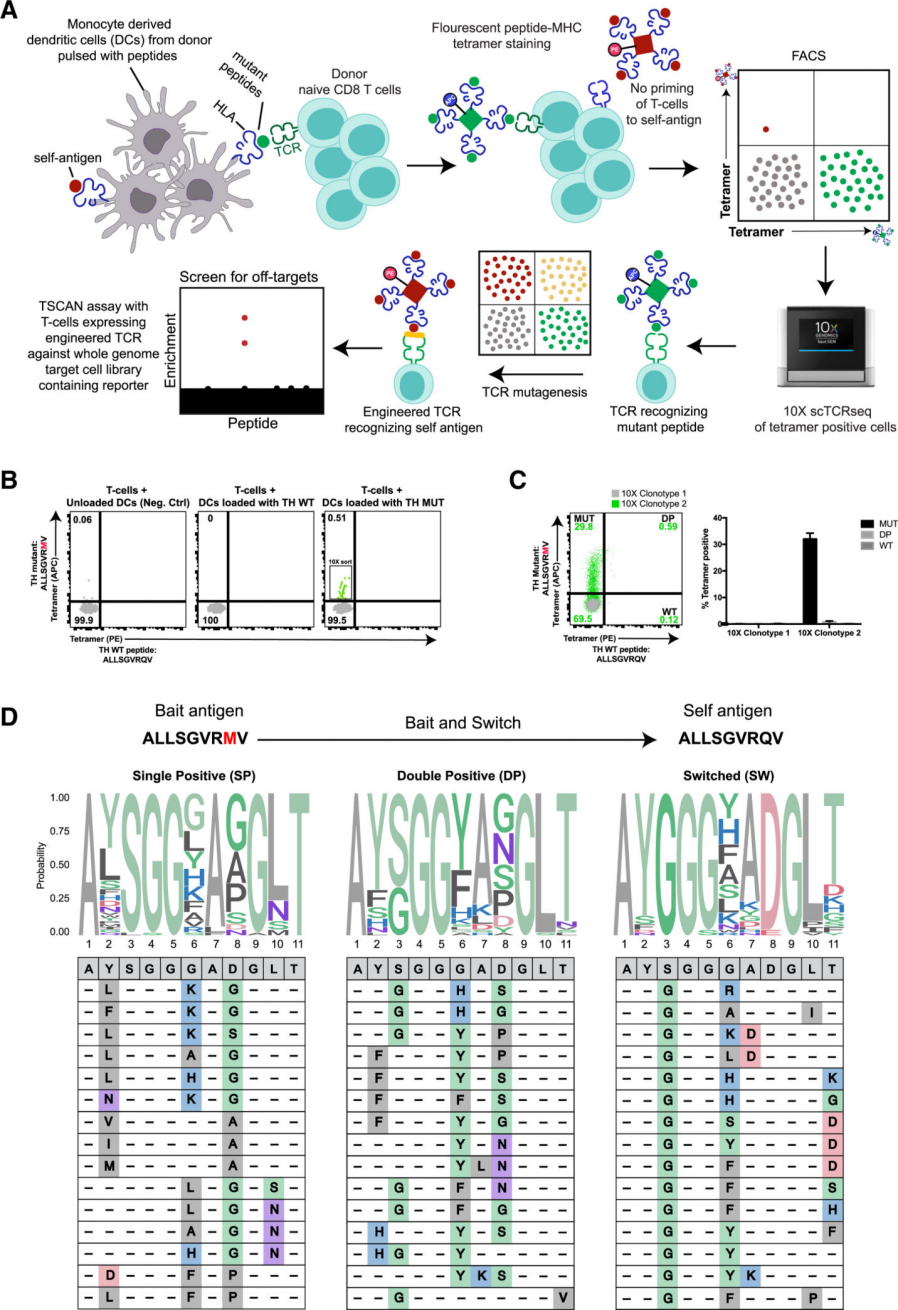
Overview of the T-Switch platform and selection of mutant peptides (A) Schematic of T-Switch workflow. Monocyte-derived dendritic cells (MoDCs) from a healthy donor are pulsed with mutant and self-peptides. Donor naive CD8 T cells will then be cultured with the antigen-presenting cells and expanded. HLA tetramer technology is used to identify T cell reactivity against any of the pulsed peptides and sorted and sent for 10X TCR sequencing to identify TCR clonotypes. CDR3 TCR mutagenesis is used to switch the specificity of the mutant-recognizing TCR to a tumor self-reactive TCR, and its off-target profile will be assessed via T-Scan. (B) Tetramer staining following antigen-specific expansion to unloaded MoDCs (left), TH WT pulsed MoDCs (middle), or TH mutant pulsed MoDCs (right). (C) Tetramer binding validation of clonotypes reconstructed from 10X TCR sequencing and expressed separately onto Jurkat TCRb^−/−^ T cells. Representative flow plot showing one replicate in left panel and bar plot in right panel showing mean ± SDs of three technical replicates. (D) Screening results for TH MUT 10X TCR CDR3α chain to switch its specificity from recognizing the bait (mutant) antigen to recognizing the self (TH WT) antigen. Amino acid sequence logo plots are generated as described in Figures 2A and 2B. Fifteen individual CDR3 sequences for each class are shown (below).

Next, we wished to translate our approach into the generation of cancer-reactive TCRs. A major advantage of our approach is that it presents a strategy for the high-throughput generation of TCRs against potential cancer targets without the need to extensively screen for T cell clones from patient samples. Instead, we can use well-established assays^30^ for *in vitro* antigen-specific activation and expansion of naive, healthy human CD8 T cells (Figure 4A). After selection of a self-antigen target, we generate mutant peptides that differ by one amino acid from the self-antigen. The goal of these neoantigen-like peptides is to bypass T cell tolerance and act as foreign peptides that can stimulate and expand naive CD8 T cells, similar to a neoantigen-like response. Monocyte-derived dendritic cells (MoDCs) from a healthy donor are then pulsed with both selected mutant and self-peptides and co-cultured with autologous naive CD8 T cells and expanded for 10 d. Next, antigen-specific T cells are identified by HLA tetramers loaded with peptides of interest. We expect to see rapid expansion to the mutant peptides as anticipated with neoantigens and no or weak expansion to self-antigens. Expanded TCR clonotypes will then be identified by 10X TCR sequencing. After identification of a TCR clonotype recognizing a mutant peptide of interest, we use that as the starting material for the generation and screening of TCR mutagenesis libraries, as described above, to switch the specificity of the TCR toward the self-antigen. We can then assess the cross-reactivity profile of the engineered self-TCRs by T-Scan against the human genome-wide library (Figure 4A). We refer to this bait-and-switch platform as T-Switch, where we bait the T cells with the mutant epitope and then switch its specificity for the self-antigen.

We applied T-Switch to the protein tyrosine hydroxylase (TH), a tumor-associated self-protein highly expressed in neuroblastomas that has relevance to neuroblastoma pathology based on published literature.^31,32^ The HLA-A2 peptide (ALLSGVRQV) from TH was selected using the HLA peptide-binding prediction program, NetMHC. To identify mutant peptides of the TH antigen, we first generated an *in silico* structure-based model of the TH peptide bound to HLA-A2 (Figure S5A). This served to visualize the “best fit” of the TH peptide in the MHC pocket and allowed us to determine the orientation of the peptide amino acids relative to the HLA molecule. Arrows pointing upward represent the amino acid side chains that face toward the TCR, and arrows pointing downward are in the direction of the MHC base (Figure S5A). An unfavorable change in energy when substituting with any amino acid at the positions pointing downward suggests that positions 2, 3, 6, and 9 may be important to maintaining the peptide in a conformation that promotes TCR engagement and are less likely to be direct interaction points for the TCR (Figures S5A and S5B). By contrast, the positions pointing upward (1, 4, 5, and 8) and having no or minimal change in energy when substituting with different amino acids are most likely TCR interaction points (Figures S5A and S5B). Another consideration in electing positions to mutate is to ensure there is no drastic change in pMHC affinity as a result. Therefore, we generated a NetMHC predicted pMHC affinity matrix for each substitution across the 9-aa peptide (Figure S5C). Based on this, we selected amino acid substitutions that (1) are in amino acids pointing upward, (2) do not significantly affect the overall stability or affinity of the predicted pMHC complex, and (3) are chemically dissimilar to the original amino acid to allow for bypass of T cell tolerance (green boxes in Figure S5C). From these mutants, we selected the glutamine to methionine mutation at position 8 (ALLSGVRMV) as our “bait” antigen. Co-culture of naive CD8 T cells against MoDCs pulsed with either the WT or mutant TH antigen showed antigen-specific T cell expansion only to the mutant peptide as measured by tetramer staining (Figure 4B). Next, we validated our *in vitro* expansion by reconstituting Jurkat TCRβ^−/−^ cells with the top two expanded clonotypes and stained the cells with tetramers loaded with either the WT peptide or the mutant peptide. The second topmost expanded clonotype bound the mutant-loaded tetramer but not the WT-loaded tetramer, as expected from the naive CD8 expansion (Figure 4C). This clonotype serves as a candidate for T-Switch to redirect its specificity toward the TH self-antigen (ALLSGVRQV).

We applied the same comprehensive library design, as before, to both the CDR3α and CDR3β chains of clonotype 2 (Figure S5D). The Jurkat TCRβ^−/−^ cell line was separately infected with each of the libraries to generate a library of T cells with variant CDR3 loops (diversity of CDR3α library: 300,000 and diversity of CDR3β library: 430,000). Antigen-specific TCRs for the SP, DP, and SW populations were isolated by tetramer staining and characterized by next-generation sequencing. Given that the bait mutation is in the C-terminal portion of the peptide, at position 8, we expected the CDR3β chain to be the primary contact to that region. Indeed, analysis of the top enriched switched TCRs of the CDR3β library revealed positions 4, 5, and 12 as key residues involved in switching specificity (Figures S6A and S6B). Validation of the CDR3β SW chain variants (Figure S6C) revealed exclusive tetramer binding to the WT TH peptide and loss of binding to the mutant peptide (Figure S6D). However, when we measured CD69 activation post co-culture of Jurkat TCRβ^−/−^ cells with target cells pulsed with the mutant or WT antigen, we found that only SW5-SW8 had functional activation (Figures S6E and S6F). This could be a result of the signaling sensitivity limits of the Jurkat cells, or it highlights the discordance between binding and activation that has been observed in previous reports.^33,34^ It is important to note that like the NLV3 CDR3β library observation of the DP TCR showing high background activation (Figure 3G), we also observed similar outcomes for the TH CDR3β DP1 and DP2 TCRs, where there was high background activation to HLA-A2 target cells without expression of the peptide tiles (Figure S6F), confirming that the DP population generates promiscuous TCRs.

**Figure 5.**
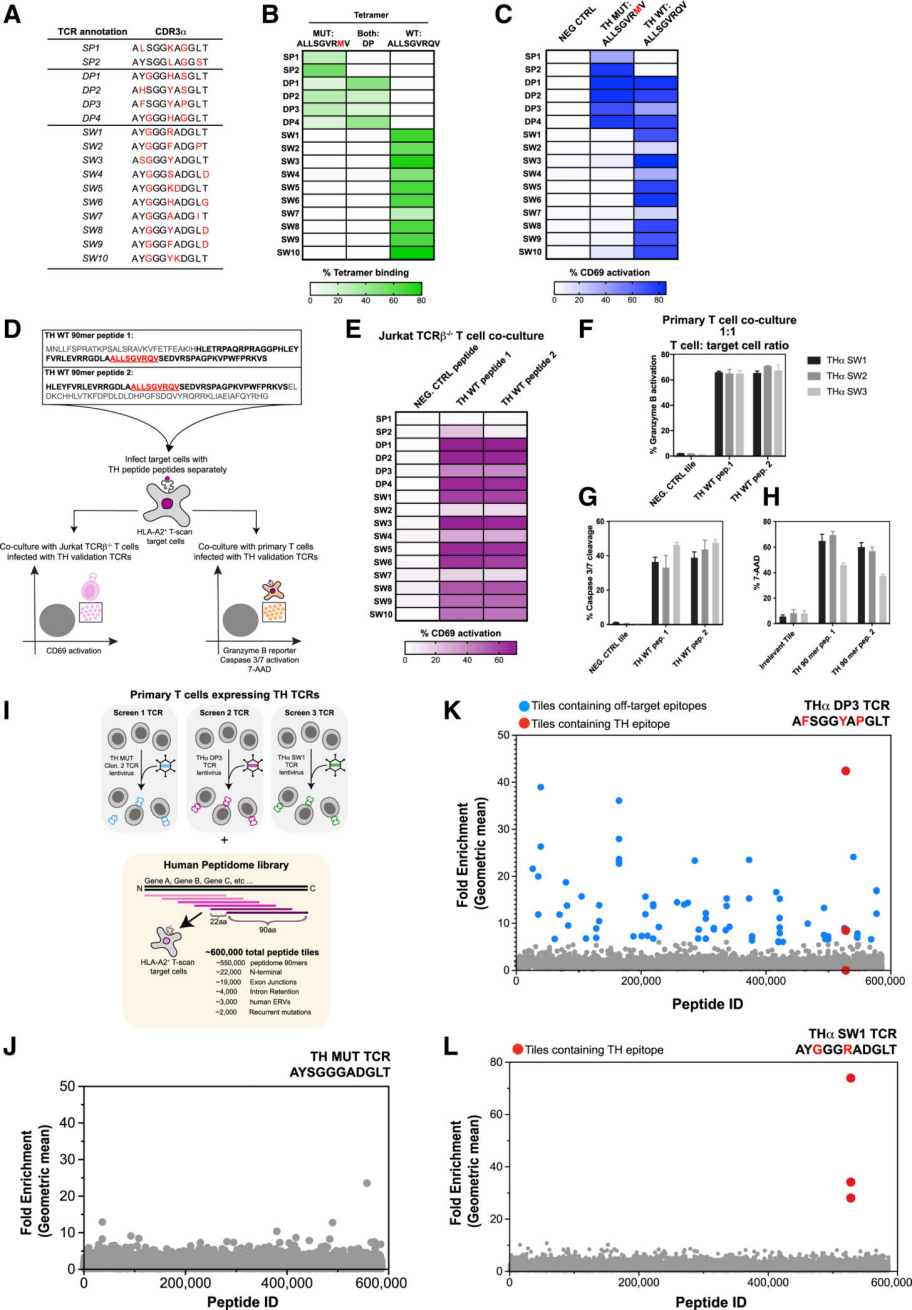
Application of T-Switch for engineering TCRs with specificity and functionality to a tumor antigen (A) TH CDR3a sequences chosen for validation experiments. (B) Tetramer binding was assessed by infecting Jurkat TCRβ^−/−^ cells infected with lentiviruses expressing the TCRs in (A). Heatmap shows summary of tetramer staining (*n* = 3). (C) CD69 activation of validation TCRs was assessed by individual 24 h co-culture of Jurkat TCRβ^−/−^ cells expressed validation TCRs with 293T-TAP^−/−^ HLA-A2 target cells pulsed with an irrelevant peptide (NEG CTRL), the mutant peptide (ALLSGVRMV), or the WT peptide (ALLSGVRQV). Heatmap shows the percentage of CD69 activation-positive Jurkat cells after co-culture as determined by flow cytometry (*n* = 3). (D) Schematic representation of functional experiments using two 90 aa peptide sequences from the TH protein expressed separately into HLA-A2+ T-scan target cells. (E) CD69 activation of validation TCRs was assessed by individual 24 h co-culture of Jurkat TCRβ^−/−^ cells expressing validation TCRs with HLA-A2+ T-scan target cells expressing a negative control peptide, the TH WT containing peptide 1 or TH WT containing peptide 2. Heatmap shows the percentage of CD69 activation-positive Jurkat cells after co-culture as determined by flow cytometry (*n* = 3). (F–H) Granzyme B activation (F), caspase-3/−7 cleavage (G), and percentage of 7-AAD positive (H) of HLA-A2+ T-scan target cells following co-culture with TH-specific TCRs. (I) Overview of human genome-wide screening strategy via T-Scan. Peptides are tiled across the human proteome in 90 aa steps with 22 aa overlaps and lentivirally expressed in HLA-A2+ T-Scan target cells. (J–L) T-Scan screen of TH MUT 10X TCR (J), TH DP TCR (K), and TH SW TCR (L) expressing T cells against the human genome-wide library. Each dot represents one peptide tile, with the y axis plotting the geometric mean of the fold change of each peptide across five replicates.

To our surprise, we found that the CDR3α chain also switched specificity. Analysis of the top enriched TCRs from each population of the CDR3α library revealed a requirement for a serine-to-glycine mutation at position three as well as a second mutation at position six to switch its specificity toward the TH WT peptide by replacing a glycine (Figure 4D). Moving a glycine from position six to position three could significantly remodel the structure of the TCR loop. This capitalizes on the complexity of antigen specificity and the need to use comprehensive directed evolution libraries to identify those rare variants. To validate our results experimentally, we reconstituted Jurkat TCRβ^−/−^ cells with the top enriched variants from each population (Figure 5A). Consistent with sequence enrichment data, T-Switch variants from the SW population of the CDR3a chain library displayed tetramer binding and CD69 activation exclusively to the TH self-antigen and not to the mutant antigen (Figures 5B and 5C). Additionally, the DP population bound and activated to both antigens, and the SP TCRs interacted exclusively with the mutant antigen (Figures 5B and 5C).

To ensure that the variant TCRs can recognize the peptide following antigen processing and presentation, we reconstituted HLA-A2+ T-Scan target cells with two 90-mer peptides that encode part of the TH protein and contain the target antigen (Figure 5D). We co-cultured the target cells with Jurkat TCRβ^−/−^ cells expressing our variant TCRs to measure CD69 upregulation or with primary T cells expressing a subset of the variant TCRs to measure target cell killing following recognition of the endogenously processed and presented antigen (Figure 5D). In response to target cells expressing the TH peptides, the engineered TCR variant expressing Jurkat cells showed potent CD69 activation, with the exception of the SP population TCRs (SP1 and SP2) that activate to the mutant TH peptide and not the WT, as expected (Figure 5E). Moreover, engineered switched variant expressing primary T cells show potent target cell killing when co-cultured with target cells expressing the 90-mer TH peptides and not when co-cultured with target cells expressing an irrelevant tile, as measured by granzyme B activation (Figure 5F), caspase-3/−7 cleavage (Figure 5G), and 7-aminoactinomycin D (7-AAD) uptake (Figure 5H). This serves as the first example of applying the T-Switch platform to redirect a TCR recognizing a bait antigen toward recognition of an endogenously processed and presented self-antigen. Therefore, the generation of additional *de novo* TCRs targeting tumor-associated self-antigens, whose production is normally prevented by tolerance mechanisms *in vivo*, is feasible with this approach.

### T-Switch variants display high specificity and mediate potent target cell killing

To examine the specificity of the engineered TCR variants and whether they exhibited cross-reactivity, we performed whole genome-wide T-Scan screens. The human peptidome library consists of ~500,000 total peptide tiles that are 90-aa fragments with 22-aa overlaps that tile across the entire human proteome (Figure 5I). The library was infected in HLA-A2 positive T-Scan target cells and co-cultured with primary T cells expressing either the original unmutated 10× clonotype that recognizes the mutant TH peptide (TH MUT TCR), the TH DP3 TCR variant with 3 mutations in the CDR3α chain, or the SW1 TH TCR variant with 2 mutations in the CDR3α chain (Figure 5I). The original TCR failed to enrich any overlapping peptide tiles, which was expected due to its recognition of the mutant TH peptide, which is not present in the library (Figure 5J; Table S5A). By contrast, the DP TCR enriched many off-target overlapping tiles from numerous self-proteins (Figure 5K; Tables S5B and S5C). Crucially, the SW TCR had strong and reproducible enrichment of 3 peptide tiles that contained our target TH WT epitope and no additional promiscuity (Figure 5L; Table S5D). This is in line with our previous data, confirming that a complete switch in specificity, following T-Switch engineering, does not introduce off-target specificities corresponding to known sequences in the human proteome. While we cannot definitively dismiss the possibility that alternative screens of cross-reactivity may uncover off-target specificities not detected here, T-Scan serves as a comprehensive and rigorous test that demonstrates the absence of unexpected toxicity within the limits of the ~600,000 90 amino acid peptides contained in the library.

### Self-antigen-reactive TCRs generated by allo-reactivity show less specificity than T-Switch

We wished to examine how the off-target profiles of the T-Switch method compare with some of the alternative methodologies used to isolate self-TCRs, such as the identification of allo-HLA-restricted TCRs by expansion of T cells with self-antigens presented in the context of foreign HLA.^35^ Since the naive T cells used have a different MHC, the TCRs recognizing the MHC-epitope combination will not have been eliminated by central tolerance. We picked three TCRs from the literature^35^ to test via T-Scan and identified multiple off-targets across the different allo-HLA-restricted TCR screens (Figures S7A–S7C; Table S6). Compared to the T-Scan screens with the SW TCR variants, all three allo-derived TCRs have a significantly higher number of off-targets, and they appear to display an intermediate phenotype in the off-target rate as compared with the screened DP and SW TCRs (Figure S7D). Therefore, we predict that allo-HLA-restricted TCRs may be inherently more cross-reactive, possibly in part by stronger recognition of the HLA molecule itself, although a larger scale analysis with additional examples will be needed to fully confirm that possible interpretation.

### T-Switch-generated anti-TH TCRs kill tumor cells expressing TH

**Figure 6.**
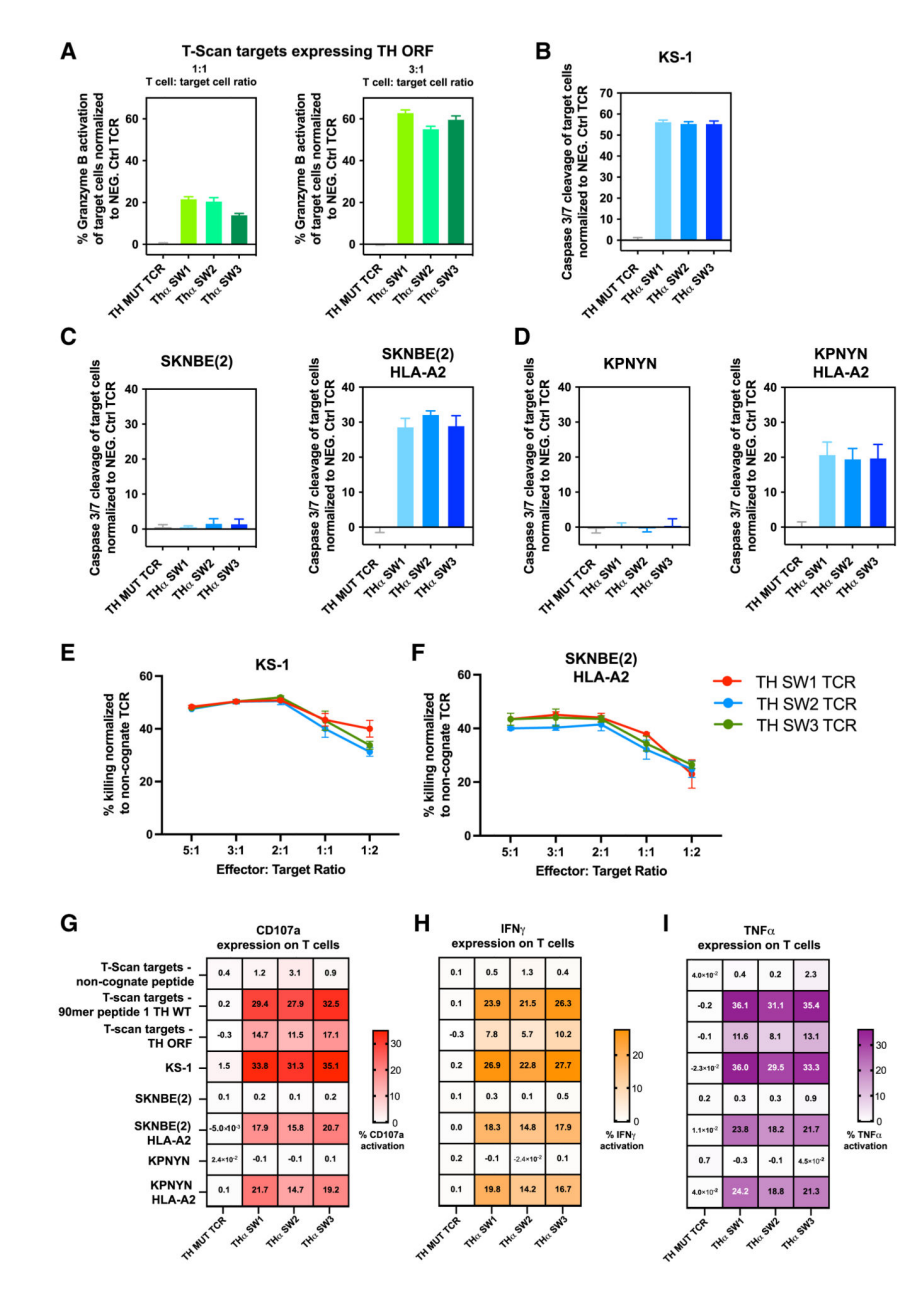
Cytotoxicity and functionality of engineered TH-specific TCR variants against TH-positive tumors (A) Granzyme B activation of 293T T-scan target cells expressing the TH full-length ORF (TH ORF) following 24 h co-culture with engineered TH WT TCR variant transduced human primary T cells or a negative control TCR at a 1:1 T cell to target cell co-culture ratio (left) or a 3:1 ratio (right). (B–D) Killing of TH-expressing cell lines by different TH-specific transduced human primary T cells. Co-culture was performed at a T cell to target cell ratio of 3:1 and cultured for 24 h. Cell lines that did not express HLA-A2 were transduced with a lentiviral vector expressing HLA-A2. (E and F) TH SW variant TCR-mediated killing, as assessed by survival of GFP transduced—KS-1 glioblastoma cells (E) and SKNBE (2) HLA-A2 neuroblastoma cells (F) after overnight co-culture with primary T cells expressing TH SW TCR variants. (G–I) Cytotoxic granule release (CD107a), IFNγ, and TNFα staining of different TH-specific TCR variant transduced primary T cells following co-culture with TH-expressing tumor cell lines. Data are normalized to a non-cognate control TCR.

We next explored whether the TH WT TCR variants could efficiently kill HLA-A2-TH^+^ tumor cells. We transduced human primary T cells with the TH MUT TCR or three SW TCR variants and first performed a co-culture with T-Scan target cells expressing the full-length TH open reading frame (ORF) (Figure 6A). Efficient granzyme B reporter activation was observed in target cells in response to co-culture with the SW TCR variants but not the TH MUT TCR (Figure 6A, left), and that response was amplified when a different effector-to-target ratio (E:T) (3:1) was used (Figure 6A, right). Next, we tested killing against three cell lines that express TH endogenously (Figure S7E). We first selected a highly expressing TH glioblastoma cell line (KS-1) that also expresses HLA-A2 and saw efficient cytotoxicity following co-culture with primary T cells expressing SW TCR variants (Figure 6B). Similar results were seen in response to neuroblastoma cell lines, such as SKNBE and KPNYN cells, which express lower levels of the TH antigen (Figures 6C and 6D). Next, we assessed the ability of TH SW TCR-T cells to target KS1 and SKNBE cells for T cell-mediated killing at multiple E:T ratios. In these experiments, we observed potent killing of target cells by primary T cells expressing the TH SW TCR variants (Figures 6E and 6F). Based on this observation, we evaluated T cell-mediated killing over time at the lowest E:T ratio of 1:2 with primary T cells expressing the TH SW TCR variants or the non-cognate TH MUT TCR co-cultured with a mixture of cognate and non-cognate cancer cells. We observed a significant loss of cognate KS1 and cognate SKNBE (2) HLA-A2 cells over time (Figure S7F). Furthermore, additional T cell metrics of functionality were also upregulated in response to co-culture with TH-expressing target cells, including the degranulation marker CD107a and cytokines interferon (IFN)-γ and tumor necrosis factor alpha (TNF-α) (Figures 6G–6I). Combined, these data show that our T-Switch variants can efficiently kill tumor cells expressing endogenously processed and presented antigen, representing therapeutic candidates.

**Figure 7.**
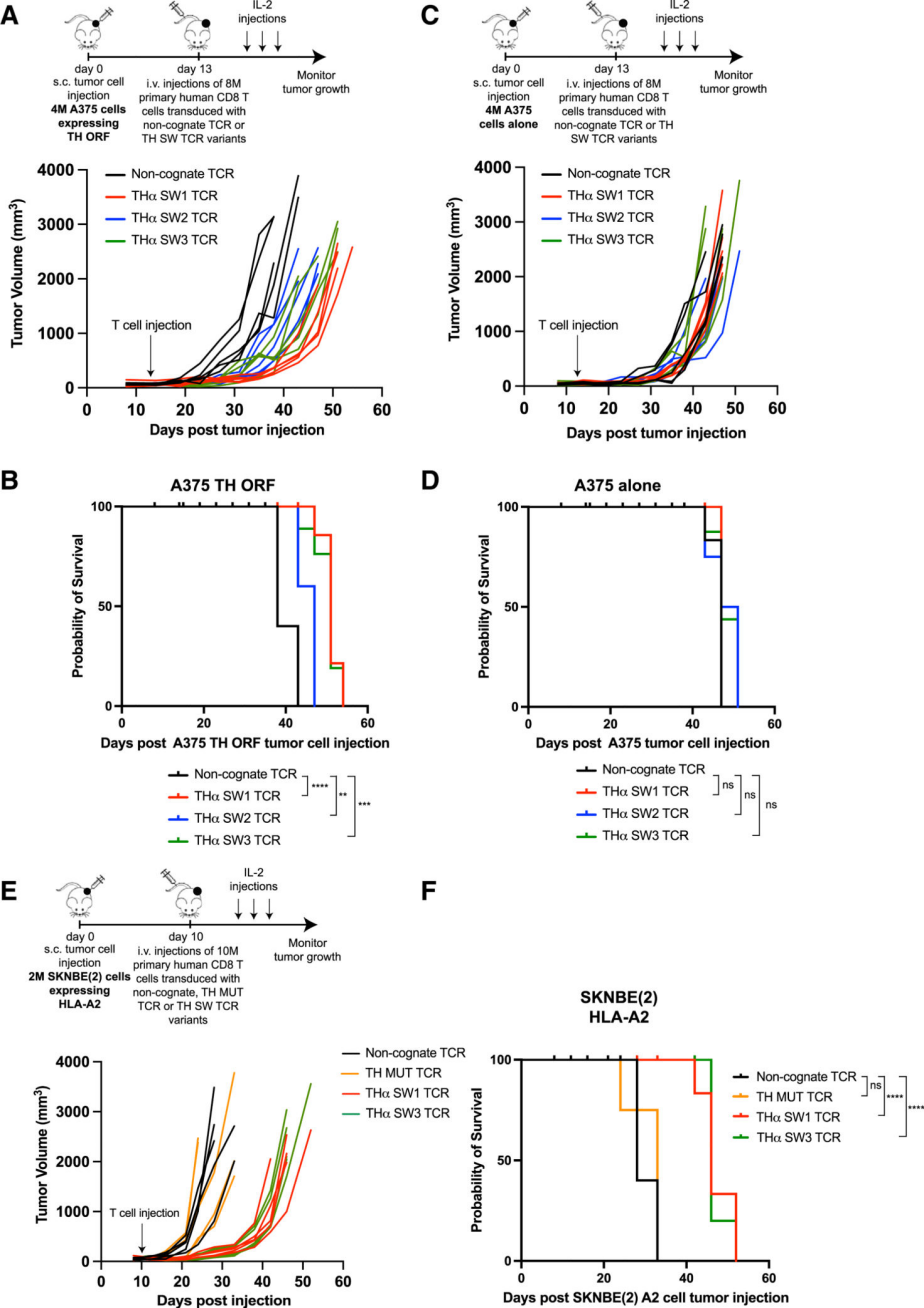
*In vivo* anti-tumor activity of TH-specific TCR variants against TH-positive xenogeneic mouse tumor models (A–D) The antitumor activity of primary T cells transduced with engineered TH SW TCRs was assessed in a xenogeneic A375 melanoma mouse tumor model. NSG mice were injected s.c. in their left flanks with 4E6 A375 cells expressing the TH ORF (A and B) or alone (C and D) on day 0 (*n* = 5), followed by the treatment with a single i.v. dose of 8E6 non-cognate TCR transduced T cells or THa SW variant TCR transduced T cells on day 13. All mice were injected periodically with s.c. injections of recombinant human interleukin (IL)-2 to promote T cell engraftment. Following treatment, mice were monitored for tumor growth and survival. Individual tumor growth curves are shown in (A) and (C). Mouse survival is displayed using Kaplan-Meier plots and compared by the log-rank (Mantel-Cox) test. Asterisks indicate significant differences; *p < 0.05, **p < 0.01, ***p < 0.001, ns, not significant. (E and F) The antitumor activity of primary T cells transduced with engineered TH SW TCRs was assessed in a xenogeneic SKNBE(2) neuroblastoma mouse tumor model. NSG mice were injected s.c. in their left flanks with 2E6 SKNBE(2) cells expressing HLA-A2 on day 0 (*n* = 5), followed by the treatment with a single i.v. dose of 10E6 non-cognate TCR transduced T cells, TH MUT, or TH SW variant TCR transduced T cells on day 10 (left) and monitored for tumor growth (left) and survival (right). Tumor growth curve and survival are displayed as in (A) to (D).

We then determined the anti-tumor activity in an A375 melanoma xenogeneic tumor model with or without expression of the TH ORF using immunocompromised NOD scid gamma (NSG) mice. A single i.v. infusion of T-Switch engineered THα SW1-SW3 TCRs showed a substantial and significant improvement in antitumor activity and survival relative to primary T cells expressing a non-cognate TCR when injected into mice bearing A375 tumor cells expressing the TH ORF (Figures 7A and 7B) but not in mice bearing A375 tumor cells alone (Figures 7C and 7D). To assess relevance to neuroblastoma, we additionally assessed anti-tumor activity in a xenogeneic SKNBE(2) HLA-A2 neuroblastoma mouse tumor model and also observed a significant reduction in tumor volume when mice are injected with primary T cells expressing our TH SW TCR variants and not when injected with a non-cognate TCR or the TH MUT TCR clonotype thatrecognizes the bait antigen (Figures 7E and 7F). This confirms that our SW variants are functional and can kill tumor cells *in vivo* that are endogenously expressing the TH protein and extend survival. Overall, our findings demonstrate how T-Switch can be used to identify synthetic TCR variants with promising therapeutic properties.

## DISCUSSION

Here, we describe the development and application of T-Switch, an engineering platform that utilizes a two-step strategy for TCR evolution to systematically and productively direct its specificity toward desired new targets, especially self-antigens. The key element of our strategy is to evolve a TCR against a neo-epitope peptide with a single amino acid change that would allow central tolerance escape. Once TCRs to the neo-epitope have been identified, we systematically mutagenize the CDR3 region of the TCR to generate a diverse library of related TCRs and then screen for binding specificity swaps from the neoantigen to the desired target. Swapping the specificity, i.e., losing the ability to recognize the initial epitope and instead recognize a new epitope, rather than gaining a new specificity, appears to be the key to avoiding deleterious cross-reactivity.

In our initial proof-of-principle experiments, we chose two viral NLV TCRs based on our previous knowledge that they have differential binding to closely related epitopes that differ by one amino acid from the immunodominant CMV epitope. Moreover, the NLV2 and NLV3 TCRs exhibit no sequence identity in their variable regions, providing us with valuable insights into the behavior of diverse TCRs on our platform. Our library design, targeted exclusively to the hypervariable CDR3 region, is systematic and comprehensive. We were able to switch specificities for both TCRs to recognize the epitopes normally only seen by the other TCR. Importantly, a major finding of our in-depth studies is that switching recognition of TCRs to peptides with only a single amino acid change requires only 2–3 amino acid changes while still retaining exquisite specificity.

Interestingly, diverse motif group enrichments in the different populations underscore the challenges of computationally predicting the determinants of TCR specificity and show that this library design lends itself well to a general approach for TCR development. Perhaps the most important lesson learned by these preliminary experiments is that switching the specificity as opposed to gaining a new specificity was a feature critical to the success of this strategy. T-Scan analysis of the NLV2α DP and the THα DP TCRs revealed recognition of multiple diverse peptides on HLA-A2, and in the case of the NLV3β DP TCR and THβ DP TCRs, we saw what could be interpreted as either improved interaction between the TCR and HLA molecule or promiscuity to a self-antigen on the target cells, two caveats typically seen by affinity-enhanced TCRs. Importantly, by engineering the switch in specificity away from the closely related epitope, we reduce the predisposition toward off-target cross-reactivity compared with affinity-matured TCRs. These small-step specificity changes are therefore essential in obtaining safe and effective TCRs.

Finally, we were able to show the therapeutic potential of T-Switch by applying it to generate TCR variants for the neuroblastoma self-antigen, TH. Surprisingly, both the CDR3α and CDR3β chains switched specificity, which shows divergence from structural data expectations and reveals the incredible plasticity of the TCR-pMHC interface, presenting a challenge for computational prediction approaches. We performed several preclinical development assays showing that engineered TCR variants expressed in primary T cells that (1) can target and kill endogenously processed and presented TH self-antigen, (2) lacked cross-reactivity with a T-Scan screen against the human proteome, (3) demonstrated potent *in vitro* tumor cell killing, and (4) showed potent and effective anti-tumor activity in *in vivo* adoptive transfer experiments.

Overall, the T-Switch platform has demonstrated the ability to change the recognition of a TCR to a distinct related antigen while maintaining its specificity, a major goal of directed T cell evolution. The strategy to circumvent self-tolerance mechanisms allows us to generalize the platform to any target of choice, which promises to have many applications in the future. For example, TCRs that recognize self are now homing agents to detect cells based on their internal contents. These could be multimerized to detect rare cell types in patients. TCRs specific for self could use their specificities as zip codes to deliver agents to specific cells. In summary, we anticipate the T-Switch platform developed here to facilitate the *de novo* generation of collections of potent and effective cancer-reactive TCRs for ACT and other future applications.

### Limitations of the study

A key limitation of the current T-Switch platform is screening/selection based on tetramer binding alone. This is problematic because of the discordance between binding and activation that we observed. Therefore, for future implementations of T-Switch, it will be important to incorporate an additional selection method based on TCR activation, such as incorporation of a nuclear factor of activated T cells (NFAT) reporter and selection based on reporter activation. Furthermore, testing of additional immortalized T cell lines will be important in overcoming the signaling sensitivity limits of Jurkat cells.

One of the main advantages of T-Switch that makes it a generalizable approach for the engineering of self-reactive TCRs is the design of neoantigens that can bypass tolerance. However, not all neoantigens are immunogenic, and the factors distinguishing immunogenic from non-immunogenic neoantigens are unclear. This limitation highlights the need to multiplex the naive expansion protocol to screen pools of mutant peptides. In that manner, we can have a higher chance of ensuring the expansion of antigen-specific T cells to at least one of the bait antigens for subsequent use in T-Switch. This also allows explorations of how the initial antigen choice impacts efficacy, whether different amino acid substitutions yield varying degrees of TCR potency, and how these changes affect the cross-reactivity profiles.

## RESOURCE AVAILABILITY

### Lead contact

Further information and requests for resources and reagents should be directed to and will be fulfilled by the lead contact, Stephen Elledge (selledge@genetics.med.harvard.edu).

### Materials availability

Information and requests for resources and reagents may be directed to the lead contact.

### Data and code availability

This paper does not report original code. Any additional information required to reanalyze the data reported in this paper is available from the lead contact upon request.

## STAR★METHODS

### EXPERIMENTAL MODEL AND SUBJECT DETAILS

#### Cell lines

Jurkat TCRβ^−/−^ (J.RT3-T3.5) cells were obtained from ATCC and cultured in RPMI (Life Technologies) with 10% FBS (Hyclone), 100 units/ml penicillin, and 0.1 mg/ml streptomycin. HEK293T (female) cells and A375 melanoma cells were obtained from ATCC and cultured in DMEM (Life Technologies) with 10% FBS, 100 units/mL penicillin, and 0.1 mg/mL streptomycin. Primary T cells were cultured in RPMI with 10% FBS, 100 units/mL penicillin, 0.1 mg/mL streptomycin, and 50 U/ml IL-2 (Sigma). Naïve CD8 T cells used for antigen-specific expansions were cultured in RPMI with 10% human serum (Sigma), 100 units/mL penicillin, and 0.1 mg/mL streptomycin. SK-N-BE(2) neuroblastoma cell line was obtained from ATCC and cultured in a 1:1 mixture of EMEM and F12 medium with 10% FBS, 100 units/mL penicillin, and 0.1 mg/mL streptomycin. The KS-1 and KP-N-YN cells lines were obtained from the JCRB Cell Bank and cultured in a 1:1 mixture of EMEM and F12 medium with 10% FBS, 100 units/mL penicillin, and 0.1 mg/mL streptomycin.

#### Primary T cell expansion

Blood collars were diluted 1:1 with PBS and slowly layered onto a Ficoll-Paque (Thermo) gradient and centrifuged at 400g for 45 mins with the brake off. The PBMC layer was extracted, washed twice with PBS and irradiated with 65 Gy irradiation. For expansions 5E6 T cells were added to 100E6 irradiated PBMCs in 100ml final volume of complete RPMI medium in addition to 50 U/ml IL-2 (Sigma), and 0.1 ug/ml anti-CD3 antibody (OKT3, eBioscience).

### METHOD DETAILS

#### TCR library design and cloning

We focused our library design on the CDR3 loop residues for either the α or β chain of each TCR, which ranged in size from 3 amino acids (aa) to 13 aa CDR3 lengths. For each CDR3 peptide, we made all single-mutant, and consecutive double- and triple-mutant, sequences scanning the whole peptide. For CDR3 lengths greater than three amino acids we limited the number of substitutions for the triple mutants as shown in Table S7A. Additionally, we included CDR3 length changes of three amino acids (GSG) appended to either the N-terminus or C-terminus of each peptide as well as all single mutant sequences scanning the whole peptide for the length changes. We reverse-translated these peptide sequences into DNA codons, making synonymous mutations when necessary to avoid restriction sites used in subsequent cloning steps (BsmBI). Each of the libraries consisted of two different nucleic acid sequences encoding the same peptide variant that served as internal duplicates. We also ensured that each peptide sequence is unique to allow for unambiguous mapping of the sequencing results by making synonymous mutations in the 50 nt at the 5’ end of the peptide sequence. We then added adaptor sequences ranging from 15–18 nt at the 5’end and the 3’end that are upstream or downstream of the CDR3 sequence and unique to each TCR to allow for Gibson cloning of the oligonucleotides. Lastly, we formed concatemers of the sequences to form the 230-nt oligonucleotide sequences that were synthesized by Agilent on a releasable DNA microarray. We PCR-amplified the DNA with primers to the adaptor sequences plus overhangs for Gibson cloning into each BsmbI-digested TCR entry backbone (See TCR design and cloning for details on backbone construction). The resulting library was then transferred into the pHAGE Ef1a DEST PGK puro lentiviral vector using Gateway cloning. At least 100x library representation was maintained during all cloning steps. Each library was packaged into lentivirus and transduced at a representation of 500x and MOI of 0.3 into Jurkat TCRβ^−/−^ cells. The cells were selected with 1ug/ml of puromycin for three days beginning at 48 h post-transduction. For each TCR library, two replicates were generated with independent infections.

#### Screening of TCR libraries

Jurkat TCRβ^−/−^ cells expressing TCR libraries were cultured at 500x and stained concurrently with tetramers loaded with either the bait antigen or the mutant antigen (the method of making pMHC tetramer is described below) and stained on ice for 30 mins. Cells were washed and consequently stained with a Pacific Blue CD3 antibody (Biologend) for an additional 30 min on ice. Each of the four different stained populations (DN, SP, DP or SW) were separately sorted into RPMI complete media to maintain cell health. DN sorted cells were pelleted and frozen, but the SP, DP and SW populations were cultured in RPMI to allow for further rounds of expansion and selection. It took 10 days to grow enough cells to continue the next round of selection. After two to three rounds of selection, cells were pelleted and submitted for next generation sequencing (NGS) (the method for NGS library preparation is described below).

#### Tetramer staining

The following peptides were synthesized and ordered through Genscript:

pp65 (NLV^WT^): **NLVPMVATV**

NLV^N495M^ (3–1): **MLVPMVATV**

NLV^V497C^ (2–1): **NLCPMVATV**

NLV^P498G^ (2–2): **NLVGMVATV**

TH WT: **ALLSGVRQV**

TH MUT: ALLSGVRMV

Peptides were loaded at 1uM onto the QuickSwitch Quant HLA-A*02:01 tetramers (PE or APC labeled) (MBL International) according to the manufacturer’s protocol. Jurkat cells were stained with tetramer at a final concentration of 1 ug/ml. Prior to staining the prepared pMHC tetramer was stored overnight at room temperature before using.

#### NGS library preparation of T-Switch screens

Cells were pelleted following sorting or expansion and gDNA was purified using the GeneJet gDNA purification kit (Thermo) according to the manufacturer’s protocol. At least 100x representation of unsorted library cells were pelleted and prepared as the input sample for each screen. All libraries were amplified in two rounds of PCR using the hot start Q5 polymerase according to the manufacturer’s protocol (Thermo). In the first PCR, 1 ug of gDNA per 100 ul reaction was amplified using primers flanking the CDR3 sequence and adding on the Illumina sequencing primer adaptors. PCR1 reactions from each replicate were pooled and 1ul was used for PCR2 in 50 ul reactions to add on the Illumina sequencing adaptors and sample specific index (PCR2_F:AATGATACGGCGACCACCGAGATCTA CACTCTTTCCCTACACGACTCCAGT; PCR2_R: CAAGCAGAAGACGGCATACGAGAT**xxxxxxx** GTGACTGGAGTTCAGACGTGT, where “**xxxxxxx**” denotes a 7-nt indexing sequence. Samples were then pooled following indexing and gel extracted using the QIAGEN Gel extraction columns according to the manufacturers protocol and run on the Illumina NextSeq using the T7-Illumina 5’primer (GGTGTGATGCTCGGGGATCCAGGAATTC).

#### TCR design and cloning

All TCRs were reconstructed using a modified version of a previously published protocol.^24^ In brief, TCR sequences were encoded as single constructs containing TCRβ P2A TCRα with the mouse TCR constant regions. Sequences were synthesized as gBlocks (IDT) and cloned into an entry vector (pENTR/D-TOPO vector) and then transferred by gateway cloning into the pHAGE EF1a DEST PGK Puro vector. TCRs obtained from 10X sequencing data were synthesized via Twist in a pENTR backbone and then transferred by gateway cloning to the pHAGE Ef1a DEST PGK puro vector.

#### Antigen-specific T cell expansion and isolation

Generation of monocyte-derived dendritic cells (MoDCs) and activation of antigen-specific T cells was carried out as previously described with minor modifications.^30^ In brief, mature MoDCs from HLA-A2 positive donors were pulsed with mutant peptides or self-antigens prior to co-culture with autologous donor naïve CD8 T cells at a ratio of 1:4 MoDCs to naïve CD8 T cells in 0.5 ml medium per well in 48-well plates in RPMI medium supplemented with 60 ng/ml of IL-21 (R&D Systems). 0.5ml of media supplemented with 5 ng/ml IL-15 and IL-7 was added to the cells three days post co-culture. Six days post co-culture cells were transferred to 12 well plates and supplemented with 1 ml of media and 5ng/ml IL-15 and IL-7. Eight days post co-culture cells were transferred to 6-well plates and supplemented with 10 ng/ml IL-15 and IL-7 and incubated for 72 h. Between days 10 and 12, co-cultures were screened for the presence of pMHC tetramer reactive CD8^+^ T cells. pMHC tetramers labeled with APC were prepared as described above. Live CD8^+^ T cells staining positively for APC-conjugated TH MUT pMHC tetramers were sorted and immediately run on the Chromium 10X controller (10X Genomics) followed by 10X single cell TCR sequencing according to the manufacturer’s protocol.

#### *In silico* modelling and affinity predictions

The *in silico* structure-based pMHC model of HLA-A2 with the TH WT antigen embedded in the MHC pocket was made using MODELLER.^39^ The program FoldX^40^ was used to model all single amino acid substitutions of the TH WT peptide and to predict each mutant’s effect on the interaction as the difference between the predicted binding energy of the MHC to the mutated and WT peptide, as follows: ΔΔG= Δ_amino acid substitution_ - Δ_WT_. ΔΔG >0 indicates that a given substitution destabilized the interaction. ΔΔG <0 indicates that a given substitution stabilized the interaction between the peptide and the MHC.

The predicted MHC binding affinity matrix was generated by substituting each amino acid to every other 19 amino acids and each peptide was run in the NetMHC prediction algorithm (https://services.healthtech.dtu.dk/services/NetMHC-4.0/). Plotted values are nM affinities based on the NetMHC algorithm.

#### T-Scan library preparation and screening

The virome-wide library was previously described.^24,41^ In brief, the library consists of 93,904 total peptide 56-aa fragments tiling across the proteomes of 206 viral species with 28-aa overlaps. The library was cloned into the pHAGE CMV NFlagHA DEST IRES puro lentiviral vector by gateway cloning. 100E6 T-Scan target cells (described previously^24^) expressing HLA-A2 were transduced with the virome library at an MOI of 1 and selected with 1ug/ml puromycin for 3 d. For the screens, 5 replicates of 100E6 target cells (1000X representation per replicate) were co-cultured with 60E6 primary T cells transduced with the NLV engineered TCR of interest (refer to T cell transduction and expansion protocol) for 8h, after which granzyme reporter (IFP)-positive target cells were sorted (Sony sorter).

The human peptidome library consisted of the human peptidome V2 library of 259,345 90-aa fragments tiling across the entire human proteome with 45-aa overlap as previously described.^24^ In addition to the V2 library, the library included a V3 library of an additional ~350,000 fragments to complement the V2 peptidome. Moreover, the library consisted of additional fragments derived from splicing defects, such as intron retention and exon junction, alternative translation products, endogenous retroviruses, antisense strands, and hypothetical proteins. This resulted in a total library size of 586,167 fragments that are 90-aa in length with 22-aa overlaps.

For the tiling mutagenesis NLV mutant libraries, the following peptides were mutagenized in the context of 56-mer and 9-mer peptides:

1. 56mer 3–1 peptide: (PWQAGILAR**MLVPMVATV**QGQNLKYQEFFWDANDIYRIFAELEG VWQPAAQPKRRR)
2. 9-mer 3–1 peptide: **MLVPMVATV**
3. 56mer 2–2 peptide: (PWQAGILAR**NLVGMVATV**QGQNLKYQEFFWDANDIYRIFAELEG VWQPAAQPKRRR)
4. 9-mer 2–2 peptide: **NLVGMVATV**

Each amino acid in bold was mutated to every other 19 amino acids. As a control the two adjacent amino acids underlined in the 56-mer peptide were also mutated to all other 19 amino acids. This set of 247 mutant peptides was combined with 171 mutant peptides from the 9-mer peptide mutants and combined with 4 versions of the WT peptide as control. Each peptide was reverse translated with non-rare human codons in two different nucleic acid sequences to serve as internal duplicates. The library was synthesized by Agilent and was amplified and sequenced as previously described.^24^

#### Primary T cell transduction

Primary CD8 T cells were isolated from apheresis collars obtained from the Brigham and Women’s Hospital Specimen Bank under protocol T0276. First, CD8 cells were isolated using the RosetteSep CD8 purification kit (StemCell) from primary blood mononuclear cells (PBMCs) according to the manufacturer’s protocol. 1E6 cells were seeded per well of a 24-well plate and stimulated with Dynabeads Human T-Activator CD3/CD28 (Life Technologies) with a 1:1 ratio. Cells were concurrently transduced with 100ul of lentivirus encoding the TCR of interest. After three days, transduced T cells were purified using biotin anti-mouse TCRβ chain antibody (Biolegend) according to the manufacturer’s protocol.

#### Co-culture assays

For CD69 validation experiments, Jurkat TCRβ^−/−^ cells expressing TCRs of interest were harvested, pelleted by centrifugation and resuspended in fresh RPMI complete media at 1E6 cells/ml. 1E5 T cells were seeded in wells of U-bottom 96-well plate. Antigen expressing cells consisted of 293T T-Scan HLA-A2 positive target cells peptide pulsed with 1uM of peptide or overexpressing the 90mer peptides containing the peptide of interest. Target cells were adjusted to 1E6 cells/ml in complete DMEM media and 1E5 cells was added to each well of the T cell plate. All validation experiments were performed in a 1:1 ratio of T cells to target cells unless otherwise noted. Co-culture samples were incubated overnight, for 16 h, at 37C, 5%CO_2_. Following co-culture, cells were co-stained with anti-human CD3 antibody (Biolegend) and anti-human CD69 antibody according to the manufacturers protocol. Following a 20-minute incubation time on ice, cells were washed twice and then assessed by flow cytometry.

For tumor killing assays, 50K KS-1, SK-N-BE(2) or KP-N-YN cells were seeded in each well of a U-bottom 96-well plate. 1.5E5 TH MUT or engineered TCR-specific human primary cells were added to each well with tumor cells and co-cultured for 48 hr. The plate was washed and then stained with the caspase 3/7 staining kit (Abcam) according to the manufacturer’s protocol and was analyzed by flow cytometry.

For primary T cell activation marker assessment, 1E5 T-Scan target cells or tumor cells were seeded in each well of a flatbottom 96-well plate and incubated overnight at 37°C. The next day 1E5 TCR-transduced human primary T cells were mixed with 1:200 anti-CD107a-PE (Biolegend) and 1:1000 brefeldin A (Biolegend) and then added to each well containing target cells. Co-culture was performed for 6 h and then the plate was washed and transferred to a 96 well U bottom plate and stained with anti-CD8-BV421. The plate was then fixed with IC fixation and permeabilized to be further stained with anti-IFN-G-BV605 (Biolegend) and anti-TNF-PECy7 (Biolegend) on ice for 30 min. The plate was then analyzed by flow cytometry.

#### Adoptive transfer of T-Switch engineered TH TCRs in xenogeneic mouse tumor models

Immunodeficient female NOD scid gamma (NSG) mice at 8 weeks of age were injected subcutaneously (s.c) with 4E6 A375 or A375 cells expressing the TH ORF in 200 ul of a 1:1 mixture of PBS and Matrigel in their left flanks. Mice were injected intravenously through tail vein injection with 8E6 non-cognate TCR transduced primary T cells or TH SW engineered primary T cells (injection volume = 200 ul) on day 13 post tumor injections. As previously described,^42^ all mice received s.c. injections of 2.75 ug recombinant human IL-2 (Millipore) on the day of T cell injection, the subsequent two days, and twice a week for the first three weeks of the experiment. Post T cell injection, tumor volume was monitored every 4 days using calipers. Tumor measurement was as follows: tumor volume = length × width^2^/2. Mice were euthanized if showing considerable weight loss (>20% of initial body weight) or if tumors grew larger than 2000 mm^3^.

### QUANTIFICATION AND STATISTICAL ANALYSIS

Statistical details of experiments can be found in the figure legends. Data analysis was performed in Python, Excel, and GraphPad Prism. All error bars in figures indicates standard deviation.

## Supplementary Material

Table S7

Key Resource Table

Supplemental Figures

Table S1

Table S2

Table S3

Table S4

Table S5

Table S6

SUPPLEMENTAL INFORMATION

Supplemental information can be found online at https://doi.org/10.1016/j.immuni.2024.11.009.

## Figures and Tables

**Table:** KEY RESOURCES TABLE

REAGENT or RESOURCE	SOURCE	IDENTIFIER

Antibodies		

CD3 antibody (OKT3)	Thermo Fisher Scientific	Cat: 14-0037-82 RRID: AB_467057
Brilliant Violet 421^™^ anti-human CD3 Antibody	Biolegend	Cat: 300434; RRID: AB_10962690
PE anti-human CD69 Antibody	Biolegend	Cat: 310906; RRID: AB_314841
APC anti-human CD3 Antibody	Biolegend	Cat: 317318; RRID: AB_1937212
PE anti-human CD107a (LAMP-1) Antibody	Biolegend	Cat: 328608; RRID: AB_1186040
PE/Cyanine7 anti-human TNF-α Antibody	Biolegend	Cat: 502930; RRID: AB_2204079
Brilliant Violet 605^™^ anti-human IFN-γ Antibody	Biolegend	Cat: 506542; RRID: AB_2801102
APC anti-mouse TCR Vβ5.1, 5.2 Antibody	Biolegend	Cat: 139506; RRID: AB_10933250
CD8-APC, human	Miltenyi biotech	Cat: 130-113-154; RRID: AB_2725982
Recombinant Human IL-21 Protein	R&D systems	Cat: 8879-IL-010
Recombinant Human Interleukin-15/IL-15	Novoprotein	Cat: P40933
Recombinant Human IL-7 Protein	R&D systems	Cat: 207-IL-010
Tyrosine Hydroxylase Monoclonal Antibody	Thermo Fisher Scientific	Cat: MA1-24654

Bacterial and virus strains		

One Shot^®^ Stbl3^™^ Chemically Competent E. coli	Thermo Fisher Scientific	Cat: C7373-03
ElectroMAX^™^ DH10B^™^ Cells	Thermo Fisher Scientific	Cat: 18290015

Biological samples		

Apheresis collars	Brigham and Women's Hospital Specimen Bank under protocol T0276	N/A

Chemicals, peptides, and recombinant proteins		

Interleukin-2, human (hIL-2)	Sigma (Millipore)	Cat: 11147528001
Gateway^™^ LR Clonase^™^ II Enzyme mix	Thermo Fisher Scientific	Cat:11791100
Gateway^™^ BP Clonase^™^ II Enzyme mix	Thermo Fisher Scientific	Cat:11789100
Q5^®^ High-Fidelity 2X Master Mix	New England Biolabs	Cat:M0492L
Lenti-X Concentrator	Takara Bio	631232
Puromycin Dihydrochloride	Thermo Fisher Scientific	Cat:A1113803
Polybrene	Thermo Fisher Scientific	Cat:TR-1003-G
Benzonase Nuclease HC, Purity > 99%	Thermo Fisher Scientific	Cat:71206-3
NEBuilder^®^ HiFi DNA Assembly Master Mix	New England Biolabs	Cat:E2621L
Dynabeads^®^ Human T-Activator CD3/CD28	Thermo Fisher Scientific	Cat:11132D
jetPRIME	Polyplus	Cat:114-07

Critical commercial assays		

QuickSwitch^™^ Quant HLA A*02:01 Tetramer Kit-APC	MBL International Corporation	Cat: TB-7308-K2
QuickSwitch^™^ Quant HLA A*02:01 Tetramer Kit-PE	MBL International Corporation	Cat: TB-7308-K1
GeneJET Genomic DNA Purification Kit	Thermo Fisher Scientific	Cat: K0722
Caspase 3/7 Staining Kit (Far Red)	abcam	Cat: ab270785
CD8+ T Cell Isolation Kit, human	Miltenyi biotech	Cat: 130-096-495
RosetteSep^™^ Human CD8+ T Cell Enrichment Cocktail	Stem Cell Technologies	Cat:15023
Anti-Biotin Microbeads Miltenyi	Miltenyi Biotec	Cat:130-090-485

Experimental models: Cell lines		

HEK293T cells	ATCC	ATCC Cat# CRL-3216 RRID:CVCL_0063
HEK293T HLA-A2 Epitope Discovery Cells (MHC KO, IFPGzB, ICADCR, HLA-A2, CASP3/7KO)	This study	N/A
SK-N-BE(2)	ATCC	CRL-2271
KPNYN	XenoTech	IFO50431
KS-1	XenoTech	IFO50436
Jurkat, Clone E6-1	ATCC	Cat:TIB-152

Oligonucleotides		

See Table S7B	-	N/A

Recombinant DNA		

NLV2 TCR CDR3 alpha library	This study	N/A
NLV2 TCR CDR3 beta library	This study	N/A
NLV3 TCR CDR3 alpha library	This study	N/A
NLV3 TCR CDR3 beta library	This study	N/A
TH TCR CDR3 alpha library	This study	N/A
TH TCR CDR3 beta library	This study	N/A
NLV epitope saturation mutagenesis library	This study	N/A
pHAGE_CMV_NFlagHA_DEST_IRES_puro	Kula et al.^24^	N/A
pHAGE_EF1a_HLA-A2	Kula et al.^24^	N/A
pHAGE-EF1: DEST-PGK: TCR constructs	This study	N/A
Human Peptidome Library	Dezfulian et al.^36^	N/A
Virome Peptidome Library	Kula et al.^24^	N/A

Software and algorithms		

Bowtie	Langmead et al.^37^	https://bowtie-bio.sourceforge.net/index.shtml
Cutadapt	Martin^38^	http://cutadapt.readthedocs.io/en/stable/
Cell Ranger v3.0.1	10X Genomics	https://support.10xgenomics.com/single-cell-gene-expression/software/pipelines/latest/what-is-cell-ranger

## Highlights

- T-Switch is an engineering strategy to direct TCR specificity toward new targets
- Specificity screening via systematic and comprehensive CDR3 mutagenesis libraries
- Switching TCR specificity is key to avoiding detrimental cross-reactivity
- T-Switch identifies therapeutic TCR variants with enhanced potency and specificity
