# Supplementary material for: T-Switch: A specificity-based engineering platform for developing safe and effective T cell therapeutics
Source: Immunity. Author manuscript; Available in PMC 2026 Jul 24. (PMC13397619; doi:10.1016/j.immuni.2024.11.009)

Supplemental Figure 1

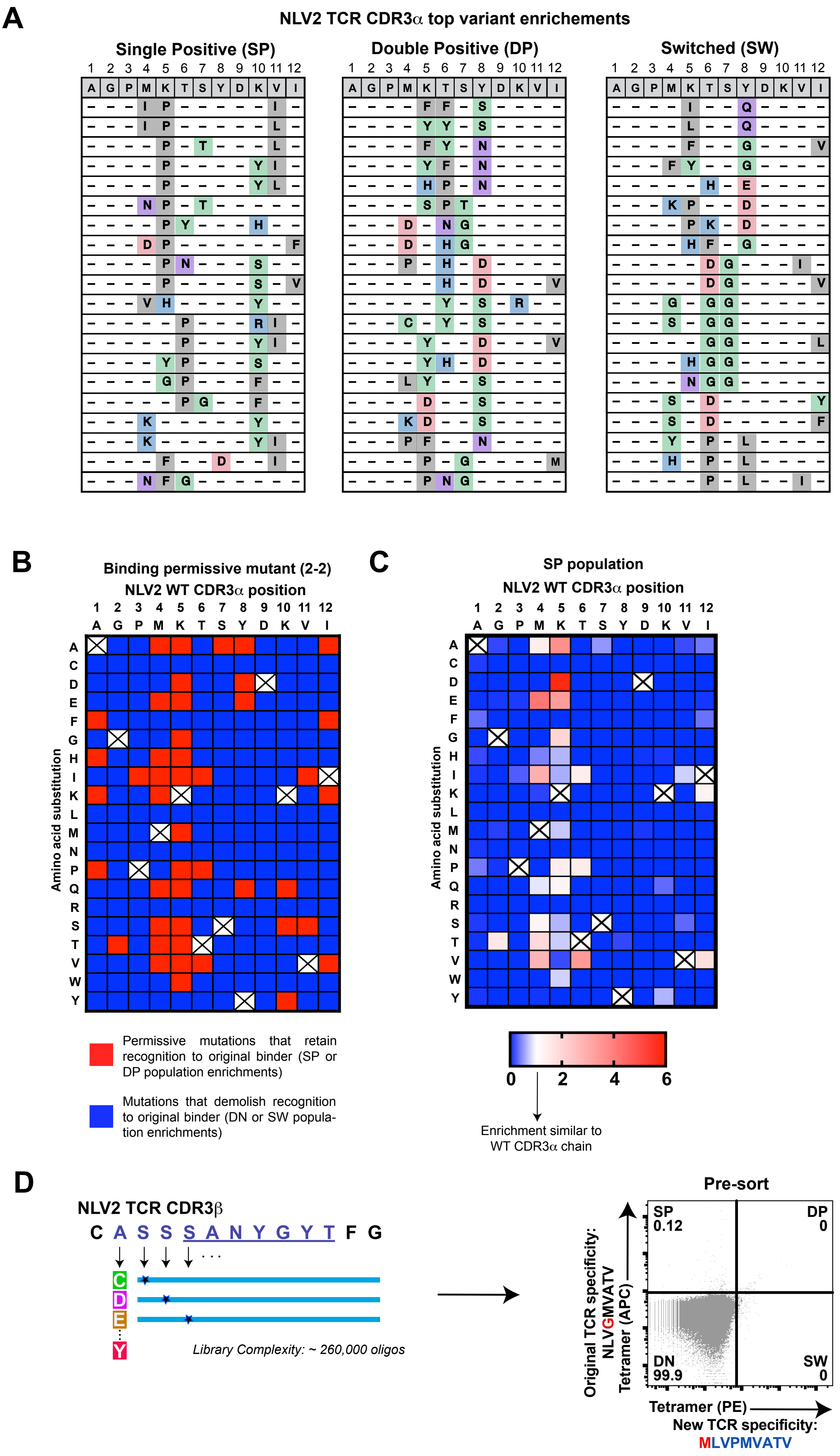

Supplemental Figure 2

A

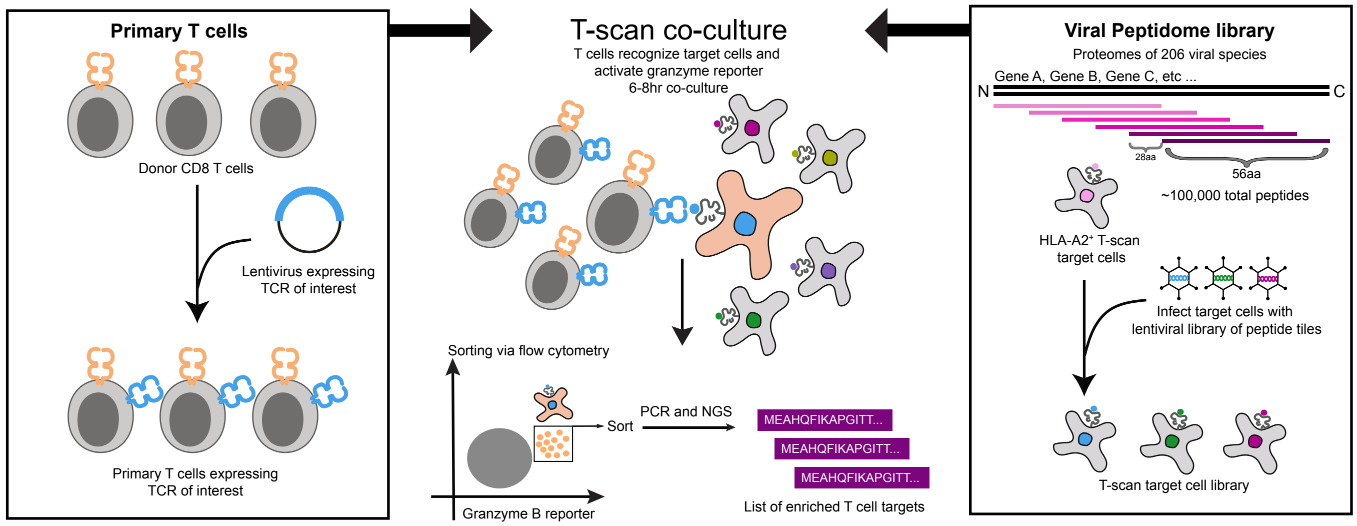

B

Cross-reactivity Profile of NLV2 DP1 TCR

	Epitope	Sequence									
Immunodominant CMV epitope	CMV:NLV^{WT}	N	L	V	P	M	V	A	T	–	V
Novel TCR peptide reactivity	CMV:NLV^{N495M}	M	–	–	–	–	–	–	–	–	–
Original TCR peptide reactivity	CMV:NLV^{P498G}	–	–	–	G	–	–	–	–	–	–
NetMHC predicted epitopes	HEVprotein$_{1749\text{-}1757}$	–	–	I	G	–	L	Q	–	–	I
	MAYVprotein$_{797\text{-}805}$	T	–	L	A	L	I	–	–	–	–
	PIV5:ProteinP$_{266\text{-}274}$	T	–	E	G	–	I	T	–	–	–
	CTFprotein$_{173\text{-}181}$	–	–	L	–	Y	I	K	–	–	L
	HCVprotein$_{1045\text{-}1053}$		–	V	G	T	I	V	–	S	L
	HCVprotein$_{1057\text{-}1065}$		–	L	G	T	I	V	–	S	L
	HHV:BBRF2$_{151\text{-}159}$	A	–	L	S	–	I	E	N	–	A
			*				*				

C

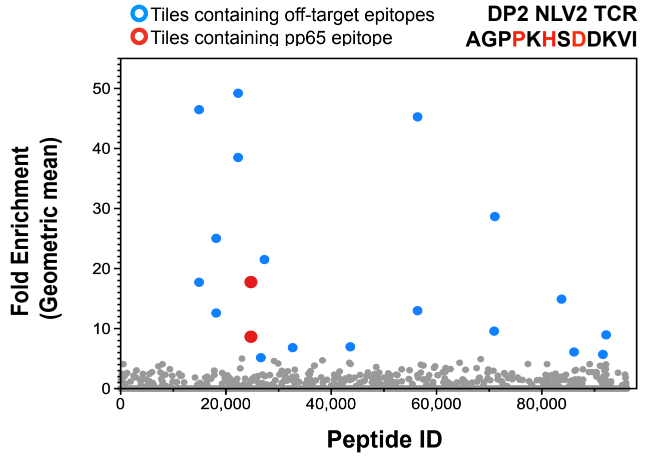

D

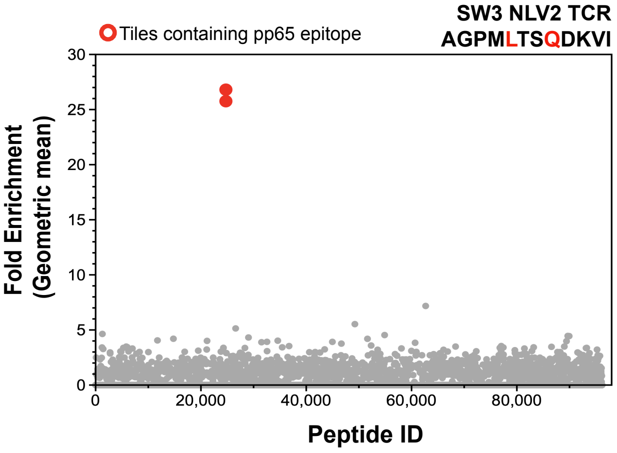

Supplemental Figure 3

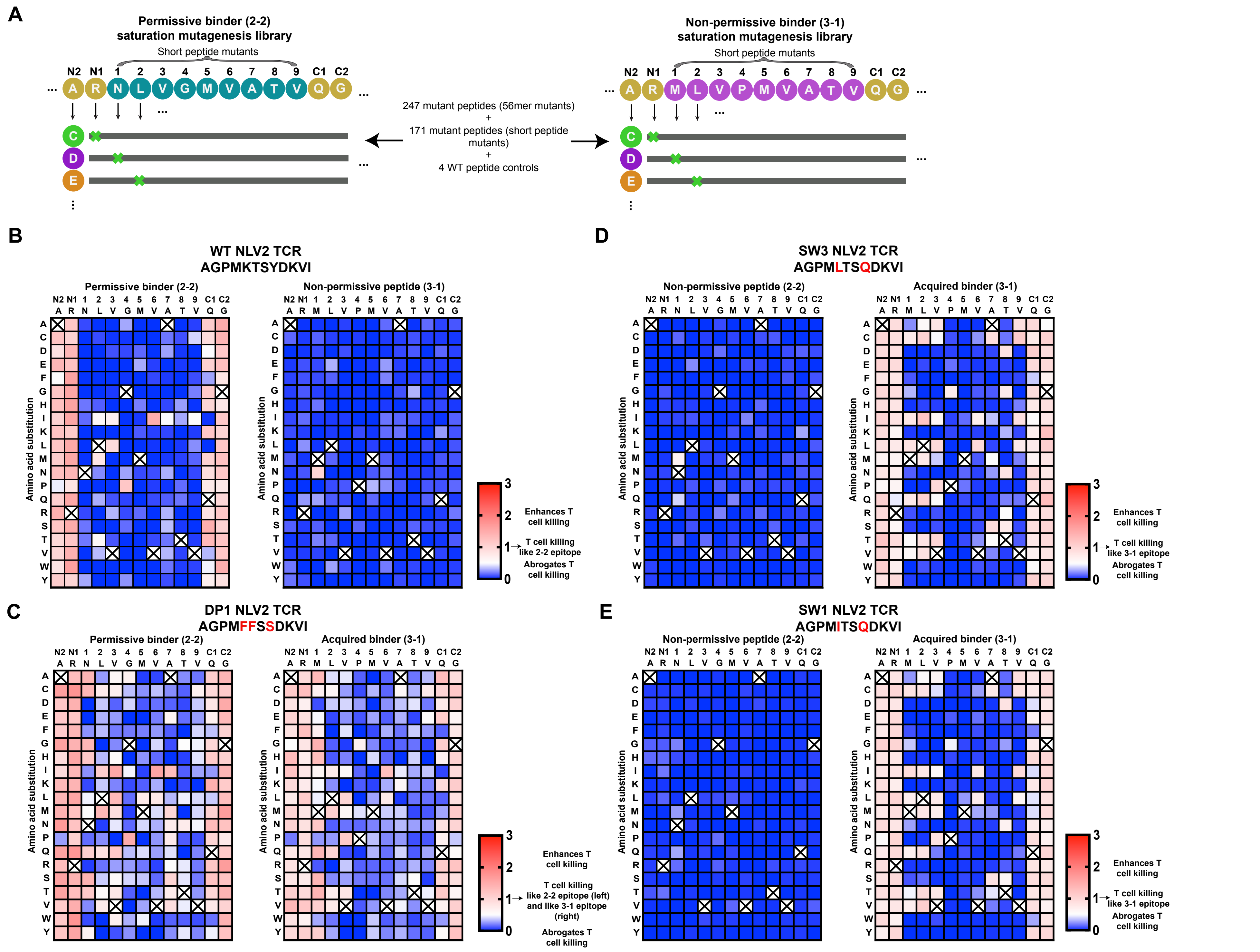

Supplementary Figure 4

A

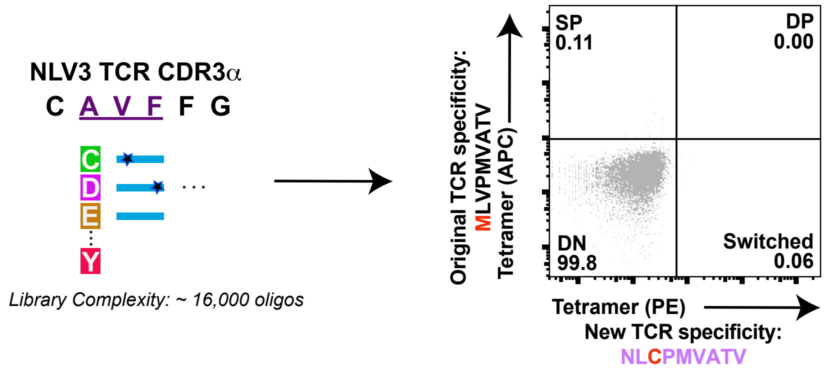

B

NLV3 TCR CDR3β top variant enrichements

Single Positive (SP)

1	2	3	4	5	6	7	8	9	10	11	12	13
A	S	S	P	T	G	G	S	P	S	P	L	H
–	–	–	–	Y	–	–	Y	–	–	K	–	–
–	–	–	–	Y	–	–	Y	–	–	D	–	–
–	–	–	–	Y	–	–	–	–	–	K	–	S
–	–	N	–	F	–	–	Y	–	–	–	–	–
–	–	–	–	Y	A	–	–	–	G	–	–	–
–	–	–	–	Y	–	–	–	–	G	–	M	–
–	–	–	–	W	–	–	–	–	G	–	–	–
–	–	–	–	Y	A	–	–	–	L	–	–	–
–	–	–	–	F	–	–	–	–	G	–	–	Y
–	–	–	–	F	–	–	–	–	G	–	–	D
–	–	–	–	F	–	–	–	–	G	–	–	N
–	–	–	–	F	–	–	–	–	G	K	–	–
–	–	–	–	I	–	–	–	–	–	E	–	–
–	–	G	–	L	–	–	–	–	–	–	–	Y
–	–	G	–	F	–	–	–	–	–	–	–	F
–	–	T	–	F	–	–	–	–	H	–	–	–
–	–	–	K	–	A	–	–	–	–	–	–	N
–	–	T	K	–	S	–	–	–	–	–	–	–
–	–	–	Q	–	–	–	–	–	–	–	–	T
–	–	–	E	W	–	–	–	–	–	–	–	–

Double Positive (DP)

1	2	3	4	5	6	7	8	9	10	11	12	13
A	S	S	P	T	G	G	S	P	S	P	L	H
–	–	–	–	L	–	–	–	A	–	–	–	–
–	–	–	–	M	–	–	–	A	–	–	–	–
–	–	–	–	V	–	–	–	A	–	–	–	–
–	–	–	–	I	–	–	–	A	–	–	–	–
–	–	–	–	L	–	–	–	V	–	–	–	–
–	–	–	–	V	–	–	–	V	–	–	–	–
–	–	T	–	L	–	–	–	S	–	–	–	–
–	–	–	–	W	–	–	–	A	–	–	–	–
–	–	–	–	Y	–	–	–	A	–	–	–	–
–	–	N	–	–	–	–	–	A	–	–	–	–
–	–	C	–	–	–	–	–	A	–	–	–	–
–	–	A	–	–	–	–	–	A	–	–	–	–
–	–	G	–	–	–	–	–	A	–	–	–	–
–	–	V	–	–	–	–	–	A	–	–	–	–
–	–	T	–	–	–	–	–	A	–	–	–	–
–	–	E	–	–	–	–	–	A	–	–	–	–
–	–	Y	–	–	–	–	–	A	–	–	–	–
–	–	–	–	–	–	–	–	A	–	–	–	–
–	–	–	–	–	–	–	–	A	–	–	–	D
–	–	–	–	–	–	–	–	A	–	M	–	–

Switched (SW)

1	2	3	4	5	6	7	8	9	10	11	12	13
A	S	S	P	T	G	G	S	P	S	P	L	H
–	–	–	–	L	Y	–	–	–	T	–	–	–
–	–	–	–	L	Y	–	–	–	L	–	–	–
–	–	–	–	L	Y	–	–	–	N	–	–	–
–	–	–	L	L	Y	–	–	–	–	–	–	–

Supplementary Figure 5

A

TH peptide A L L S G V R Q V

Predicted HLA-A2 affinity (NetMHC) = 33nM

In silico modelling

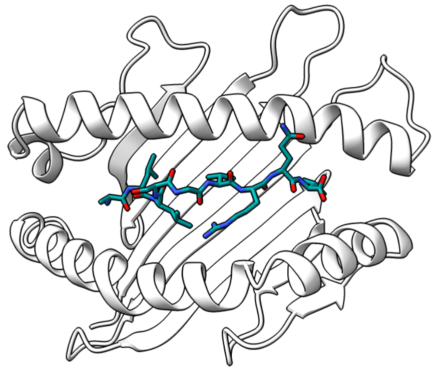

B

Predicted pMHC FoldX energy (ddg)

Amino acid substitution	1 A	2 L	3 L	4 S	5 G	6 V	7 R	8 Q	9 V
A		2.8	2.1	-0.1	0.0	1.3	0.8	0.2	0.2
C	0.9	2.8	2.0	0.0	0.0	1.1	0.0	-0.4	0.0
D	2.3	4.0	2.2	-0.2	0.0	1.4	-0.3	1.1	3.4
E	1.0	3.4	2.7	-0.3	0.1	0.5	0.5	0.1	4.3
F	-0.9	2.1	-0.1	-0.1	0.2	0.6	-0.8	-1.2	13.7
G	0.7	4.9	5.6	-0.1		2.2	1.4	0.9	1.4
H	-0.9	2.1	1.3	0.0	0.1	13.6	0.3	0.1	11.5
I	-0.6	3.5	0.4	-0.6	-0.1	0.2	-1.6	-1.0	-0.2
K	-0.6	4.6	2.4	-0.1	0.3	0.6	-0.4	-1.0	4.5
L	-1.6			-0.1	0.0	-0.5	-0.5	-1.4	-1.6
M	-1.3	0.0	0.0	0.0	-0.4	1.7	-0.8	-0.6	-0.9
N	0.1	2.9	1.5	0.0	0.0	2.3	0.9	0.4	2.2
P	4.9	5.1	8.1	-1.3	-0.1	0.2	-0.3	-0.4	4.6
Q	0.6	2.5	1.9	0.3	0.1	2.5	0.3		0.2
R	-0.2	5.4	2.7	-0.1	0.1	2.7		-0.9	5.3
S	1.7	3.3	2.3		0.1	2.1	1.2	0.1	2.2
T	1.7	4.4	2.7	0.1	-0.2	1.7	0.0	0.3	1.2
V	-0.1	3.5	1.0	0.3	0.2		-0.7	-0.7	
W	-2.2	9.6	0.0	-0.7	0.1	17.8	-0.5	-0.9	15.4
Y	-0.2	3.7	0.2	-0.1	0.2	22.9	-0.7	-0.9	5.5

Scale: 0, 10, 20

C

Predicted MHC affinitiy (NetMHC)

Amino acid substitution	1 A	2 L	3 L	4 S	5 G	6 V	7 R	8 Q	9 V
A		4952	77	35	52	132	7	19	413
C	119	11168	292	39	31	58	7	31	6445
D	4301	23622	116	13	52	227	12	93	25686
E	1345	21746	1283	15	66	251	10	13	22191
F	6	10948	20	84	23	41	3	10	6930
G	44	19360	413	41		379	30	20	13455
H	90	24155	271	53	38	352	6	25	22311
I	26	390	41	204	35	22	6	59	146
K	20	21969	2434	85	75	756	38	40	20118
L	28			165	38	25	7	19	121
M	12	29	12	96	33	35	5	26	618
N	103	22401	238	39	64	138	9	24	22678
P	3823	22751	332	34	140	110	4	9	15739
Q	118	744	326	88	79	203	9		18598
R	46	23818	2555	120	104	1040		35	19165
S	30	8042	151		52	102	9	15	8734
T	52	2070	277	50	67	55	7	18	2266
V	38	987	143	165	42		7	45	
W	27	13401	17	45	15	92	3	16	13163
Y	6	18157	28	51	15	87	4	8	14710

Scale: 0, 500, 1000, 1500, 2000

D

1. TH MUT TCR CDR3α (Clonotype 2)

C A Y S G G G A D G L T F G

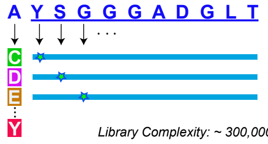

Library Complexity: ~ 300,000 oligos

DNA oligonucleotide mutagenesis library generation
Single mutants
Double mutants
Subset of triple mutants
CDR3 length changes (+/- GSG in N or C terminus)

2. TH MUT TCR CDR3β (Clonotype 2)

C S V E L Q T A P I D G Y T F G

Library Complexity: ~ 430,000 oligos

↓

Infection of Jurkat TCRβ-/- cells

↓

Select antigen-specific TCRs with MHC tetramers

↓

Deep sequencing to charcterize variant TCRs

Supplementary Figure 6

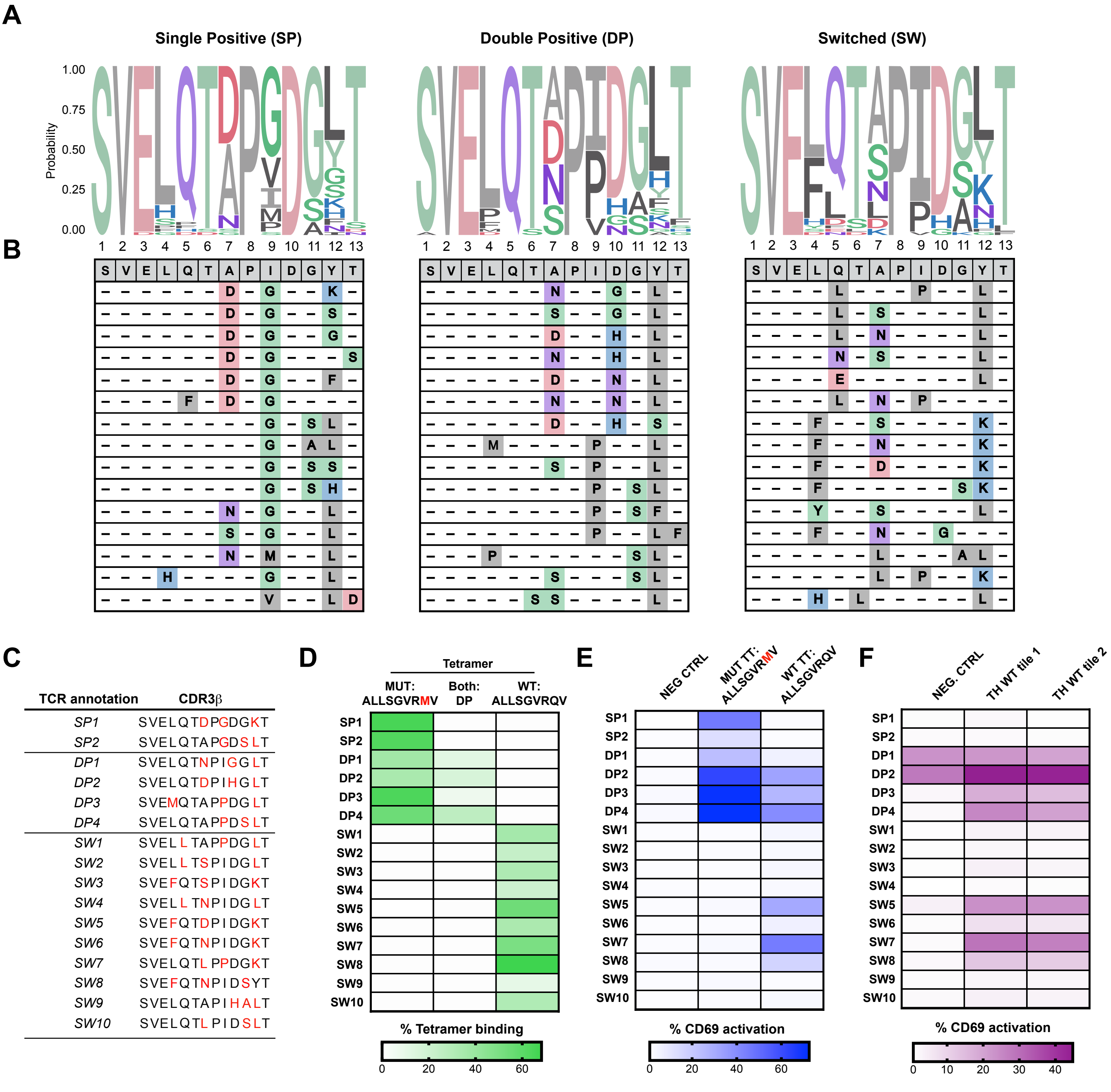

TCR annotation	CDR3β
SP1	SVELQTDPGDGKT
SP2	SVELQTAPGDSLT
DP1	SVELQTNPIGGLT
DP2	SVELQTDPIHGLT
DP3	SVEMQTAPPDGLT
DP4	SVELQTAPPDSLT
SW1	SVELLTAPPDGLT
SW2	SVELLTSPIDGLT
SW3	SVEFQTSPIDGKT
SW4	SVELLTNPIDGLT
SW5	SVEFQTDPIDGKT
SW6	SVEFQTNPIDGKT
SW7	SVELQTLPPDGKT
SW8	SVEFQTNPIDSYT
SW9	SVELQTAPIHALT
SW10	SVELQTLPIDSLT

Supplementary Figure 7

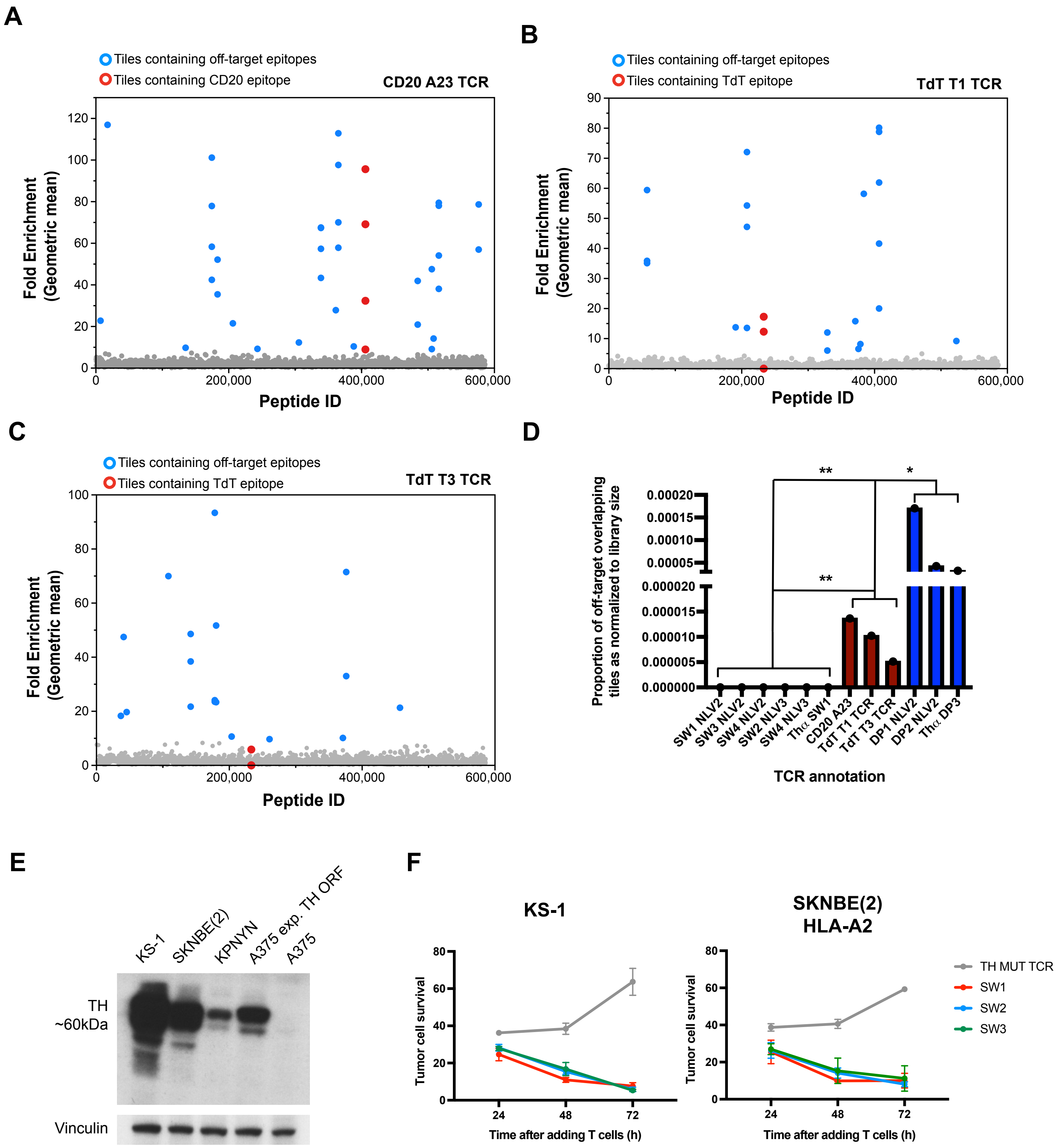

Supplement: Supplemental Figures [file NIHMS2082099-supplement-Supplemental_Figures.pdf]
